# Vitamin E hydroquinone is an endogenous regulator of ferroptosis via redox control of 15-lipoxygenase

**DOI:** 10.1371/journal.pone.0201369

**Published:** 2018-08-15

**Authors:** Andrew Hinman, Charles R. Holst, Joey C. Latham, Joel J. Bruegger, Gözde Ulas, Kevin P. McCusker, Akiko Amagata, Dana Davis, Kevin G. Hoff, Amanda H. Kahn-Kirby, Virna Kim, Yuko Kosaka, Edgar Lee, Stephanie A. Malone, Janet J. Mei, Steve James Richards, Veronica Rivera, Guy Miller, Jeffrey K. Trimmer, William D. Shrader

**Affiliations:** BioElectron Technology Corporation, Inc., Mountain View, California, United States of America; University of Pittsburgh, UNITED STATES

## Abstract

Ferroptosis is a form of programmed cell death associated with inflammation, neurodegeneration, and ischemia. Vitamin E (alpha-tocopherol) has been reported to prevent ferroptosis, but the mechanism by which this occurs is controversial. To elucidate the biochemical mechanism of vitamin E activity, we systematically investigated the effects of its major vitamers and metabolites on lipid oxidation and ferroptosis in a striatal cell model. We found that a specific endogenous metabolite of vitamin E, alpha-tocopherol hydroquinone, was a dramatically more potent inhibitor of ferroptosis than its parent compound, and inhibits 15-lipoxygenase via reduction of the enzyme’s non-heme iron from its active Fe^3+^ state to an inactive Fe^2+^ state. Furthermore, a non-metabolizable isosteric analog of vitamin E which retains antioxidant activity neither inhibited 15-lipoxygenase nor prevented ferroptosis. These results call into question the prevailing model that vitamin E acts predominantly as a non-specific lipophilic antioxidant. We propose that, similar to the other lipophilic vitamins A, D and K, vitamin E is instead a pro-vitamin, with its quinone/hydroquinone metabolites responsible for its anti-ferroptotic cytoprotective activity.

## Introduction

Ferroptosis is a form of iron-dependent, lipid oxidation-mediated programmed cell death implicated in a wide array of disease pathologies including inflammation, ischemia-reperfusion injury, and neurodegeneration [1–5]. Vitamin E modulates ferroptosis via regulation of the oxidoreductase enzyme 15-lipoxygenase (15-LO) through an unknown mechanism [6–8]. The ascribed clinical benefits of vitamin E include regulation of oxidative stress, inflammation, and cell death, each of which results from ferroptosis [1]. To elucidate the biochemical mechanism of vitamin E activity, we systematically investigated its major vitamers and metabolites, their effect on 15-LO, and the regulation of ferroptosis. We report that an endogenous metabolite of vitamin E, alpha-tocopherol hydroquinone, is a potent inhibitor of ferroptosis through its reduction of the non-heme iron in 15-lipoxygenase from its active Fe^3+^ state to its inactive Fe^2+^ state. We propose that the anti-ferroptotic effect of vitamin E results from a four-step biochemical mechanism: 1) oxidative hydrolysis of alpha-tocopherol to alpha-tocopherol quinone; 2) reduction of alpha-tocopherol quinone to alpha-tocopherol hydroquinone; 3) inhibition of 15-LO via reduction of its non-heme Fe^3+^ center to the inactive Fe^2+^ state by alpha-tocopherol hydroquinone; and 4) inhibition of the ferroptosis cascade by blocking formation of lipid peroxidation products. These results support a reinterpretation of the mechanism of vitamin E, which has previously been attributed to a non-specific antioxidant activity. As with the other fat-soluble vitamins (A, D, and K), our data suggest that vitamin E is a pro-vitamin that is converted to an active form, vitamin E hydroquinone, which functions at a specific set of targets. These findings link vitamin E’s purported clinical effects to an anti-ferroptotic mechanism-of-action.

## Materials and methods

### General method for the chemical conversion of a vitamin E chroman to a vitamin E quinone

To a round-bottomed flask, the chroman (1 eq, approximately 0.5 g) was dissolved in a 2:1 acetonitrile:water mixture (15 mL). This solution was cooled to 0°C. A solution of ceric ammonium nitrate (2.1 eq) in water (5 mL) was added dropwise. The reaction mixture was stirred at 0°C for 2 h. Upon reaction completion, the mixture was neutralized with sat. aq. NaHCO_3_ and extracted 3x with ethyl acetate. The organic phase was dried over Na_2_SO_4_ and concentrated *in vacuo*. The residue was purified by silica gel chromatography to obtain pure product.

### General method for the chemical conversion of a vitamin E quinone to a vitamin E hydroquinone

To a 20 mL septum-capped vial, the vitamin E quinone (1 eq, approximately 0.5 g) was dissolved in tetrahydrofuran (THF) (15 mL). Then the vessel was purged with H_2_. Lindlar catalyst (for the α-, γ- and δ-tocotrienol quinone) or 10% Pd/C (for the α-, γ- and δ-tocopherol quinone) (0.1 eq by weight) was added and the vessel was purged again with H_2_. The resulting suspension was stirred at room temperature overnight under a H_2_ atmosphere. Upon reaction completion, celite was added. The resulting slurry was filtered through a fritted funnel. The filter cake was washed successively with THF. The filtrate was concentrated *in vacuo* to obtain pure product.

### Cells and cell culture

Mouse striatal Q7 cells (ST*Hdh*^Q7/Q7^; immortalized by SV large T antigen) were obtained from Coriell. Cells were cultured in high-glucose (25 mM) DMEM (Gibco) medium supplemented with fetal bovine serum (10% v/v; Sigma), penicillin (100 U/mL; Gibco), and streptomycin (100 μg/mL; Gibco). For routine maintenance, Q7 culture medium also contained Geneticin Sulfate (G418; Santa Cruz Biotechnology). Cells were routinely passaged every 3–4 days, maintaining sub-confluent cell densities.

#### Cellular survival assay

Ferroptotic cell death was induced in Q7 cells using RSL3, a GPX4 inhibitor previously described [9]. All four stereoisomers of RSL3 were synthesized and purified using published methods. All subsequent cell treatment experiments utilized the single stereoisomer, (1*S*,3*R*)-RSL3 that most potently (IC_50_ ~200 nM) inhibited Q7 cell viability. To assess compound rescue activity and potency from ferroptotic cell death, Q7 cells were seeded in clear bottom, black wall tissue culture-treated polystyrene plates (Corning) using either an electronic multichannel pipette or a Multidrop™ Combi Reagent Dispenser (ThermoFisher Scientific). Cells were incubated for 5 hours at 33°C in an atmosphere with 95% humidity and 5% CO_2_ to allow attachment of the cells to the culture plate. Test compounds were administered to the desired final concentrations using a D300e Digital Dispenser (Tecan), followed within 15 minutes by RSL3 (2 μM final concentration). DMSO diluent was back-filled to a final concentration of 0.3% (v/v). Cell plates were incubated at 33°C (5% CO_2_, 95% humidity) for 18 hours. After a 15-minute equilibration to room temperature, cell number/viability was assessed using the CellTiter-Glo^®^ 2.0 Cell Titer Glo 2.0 reagent (a luminescence-based assay to quantitate ATP; Promega) which was added using the Multidrop™ Combi Reagent Dispenser (Thermo Fisher Scientific). After 15 minutes of incubation at room temperature, the luminescence (100 ms integration time) per well was determined using the BioTek Synergy plate reader. Data was imported into and analyzed using Dotmatics Studies software suite. EC_50_ values for each compound were estimated using standard four-parameter curve fitting algorithms. pEC_50_ is defined as negative-log_10_ (EC_50_) in M units. RSL3 treatment typically reduced cell number/viability by >80%. The assay performance was assessed by Z-prime calculations on each assay plate, with observed Z-prime values of >0.7. We also note that maximally efficacious concentrations of the vitamin E compounds restored RSL3-treated cell viability to levels comparable to cells that had not been treated with RSL3, indicating complete rescue of cell viability by these compounds.

### Cellular lipid oxidation assay

Ferroptosis was induced by RSL3 (2 μM final concentration) in Q7 cells as described above. The protective activity of co-treated compounds was assessed also as described above. The rate of cellular lipid oxidation after GPX4 inhibition was assessed by monitoring the time-dependent changes in green fluorescence of cells pre-labeled with BODIPY™ 581/591 C11 dye (5 μM, 30-minute labeling period; Thermo Fisher/Invitrogen) using the IncuCyte S3 live-cell imaging apparatus (Sartorius) using a 10x objective. Cells (three images per field; at least three replicate wells per condition) were imaged once hourly for up to 18 hours after compound and RSL3 addition. Quantification of cell images was performed using the IncuCyte Zoom software using the Basic Analyzer tool which utilized an algorithm that calculated the total green-positive area per well where “green-positive” was defined as 1.45 times the local background-subtracted fluorescent signal. After a 2-3-hour lag period post-RSL3 treatment, the cellular green fluorescence signal began increasing. The rate of change in total green-positive area per well from 3–9 hours post-compound treatment was calculated, then expressed relative to that observed in the RSL3-only control treatment group, which was defined as 100%.

### *In vitro* intra-cellular quantification of alpha-tocopherol (αT), alpha-tocopherol quinone (αTQ) and alpha-tocopherol hydroquinone (αTHQ) concentrations

Q7 cells were cultured in standard culture medium for 18 hours prior to compound addition. After the specified treatment time, the culture medium was removed and adherent cells were washed with PBS. The wash was removed and adherent cells were harvested by adding 1 mL of 100 mg/mL succinic anhydride in 95:5 (acetonitrile:triethylamine) followed by immediate cell scraping. After cell scraping, samples were transferred to a 1.5 mL Eppendorf tube, vortexed for 15 seconds, and kept on wet ice until all samples were harvested. Samples were vortexed again for 15 seconds and centrifuged in a Thermo Sorvall Legend XFR centrifuge at 12,000 rpm, 4°C, for 3 minutes.

Following centrifugation, samples were protein-precipitated by addition of 3 volumes of acetonitrile. Following precipitation, samples were vortexed for 30 seconds and centrifuged in a Thermo Sorvall Legend XFR centrifuge at 4000 rpm, 4°C, for 10 minutes. Subsequently, 1 volume of supernatant was transferred to a 384-well injection plate and diluted with one volume of mobile phase A and pipette mixed. Internal standard was added to the 384-well plate and pipette mixed. The injection plate was vortexed and centrifuged for a final time at 4000 rpm, 4°C, for 10 minutes and subsequently placed in a chilled Shimadzu SIL-30AC autosampler at 4°C for LC-MS/MS analysis.

Samples were analyzed using a developed method on a Shimadzu 30AD using micro-flow with a Shimadzu SIL-30AC autosampler, and coupled to an AB Sciex 6500+ QTRAP mass spectrometer. The MS/MS instrument was operated in positive ESI mode. Electrospray conditions for the μLC-ESI-MS/MS method were optimized and were as follows: Ion spray voltage was set to 5500V, temperature of 250°C, curtain gas of 10, CAD gas of 10, and ion source gas 1 and 2 of 15 and 60 psi respectively. Analyzer parameters were optimized for each compound using a combination of manual tuning and compound optimization. The following MRM transitions were used to quantify concentrations of **αT**, **αTQ** and **αTHQ**. **αT**: 430.257→165.1; **αT**-succinate: 531.368→265.3; **αTQ**: 429.253→165.1; **αTHQ**: 431.253→165.1; **αTHQ**-mono succinate: 572.208→554.1; **αTHQ**-bis succinate: 666.458→531.4. For concentrations reported: **αT** = [**αT**] + [**αT**-succinate]; **αTQ** = **αTQ**; **αTHQ** = [**αTHQ**] + [**αTHQ**-mono succinate] + [**αTHQ**-bis succinate]. Note: **αTHQ** and **αTHQ**-mono succinate were not observed in this experiment.

A Phenomenex Kinetex C_18_ (2.1 x 50 mm, 1.3 μm) column using a reverse phase gradient mobile phase method was used for chromatographic separation. The composition of mobile phase A was 0.1% formic acid in water. The composition of mobile phase B was 3:1 (0.1% formic acid in (acetonitrile: isopropyl alcohol)). Mobile Phase B was ramped from 30% to 98% over 7 minutes. The total run time was 8 minutes. Data were analyzed using Sciex Analyst Chromatography software, version 1.6.2 and Sciex MultiQuant software. The standard curve equation (y = mx +b) is generated from the calibration standards with weighted regression. Our recent PCT Publication No. WO 2018/081644 also describes the hydroquinone succinate capping methodology in more detail. Intracellular concentrations were estimated assuming spherical cells with an average diameter of 18 μm (estimated using Coulter impedance measurement), with input of 800,000 cells per 1 mL extraction volume.

### d_4_-α-Tocopherol (d_4_-αT) *in vivo* biodistribution and quinone conversion study

This study was approved by the MPI Research IACUC Committee. Animal treatment was in compliance with the U.S. Department of Agriculture’s (USDA) Animal Welfare Act (9 CFR Parts 1, 2 and 3) and performed in an AAALAC accredited facility (File #000243) following The Guide for the Care and Use of Laboratory Animals, Institute of Laboratory Animal Resources, National Academy Press, Washington, D.C. The facility maintains an Animal Welfare Assurance statement with the National Institutes of Health Office of Laboratory Animal Welfare.

Fed male CD^®^ Sprague-Dawley rats (aged ~6 weeks, average animal weight 183 g; n = 4) were dosed once daily for four days by oral gavage to achieve 100 mg/kg dose of d_4_-α-tocopherol formulated in sesame oil. Four hours after final dose, 0.5 mL of whole blood was collected into Lithium Heparin tubes via the sublingual vein. Blood was centrifuged within 15 minutes of collection and clear supernatant was transferred to 0.2 mL cryotubes. Plasma samples were rapidly frozen and then stored at -80°C until analysis. Euthanasia was performed by carbon dioxide inhalation, after which the tissues were collected and weighed, then chilled PBS was added at a ratio of 3 mL per gram of tissue for homogenization. The homogenate was frozen on dry ice and stored at -80°C until further analysis.

Quantitation of d_4_-α-tocopherol and d_4_-alpha-tocopheryl quinone in plasma and tissues was performed using an Eksigent microLC 200 coupled to a Sciex 6500 Qtrap. Briefly, samples were thawed at 4°C then vortexed. Both plasma and tissue homogenates were protein-precipitated by the addition of ice cold acetonitrile in a 3:1 ratio (ACN:sample). Following precipitation, samples were vortexed for 30 seconds and centrifuged at 4000 rpm for 10 minutes at 4°C. Fifty microliters of supernatant was added to a PCR injection plate containing 50 μL of a 0.1% Formic Acid solution then sealed for mass spectrometry analysis.

d_4_-α-Tocopherol and d_4_-α-tocopheryl quinone were baseline resolved on a Waters BEH-C_18_ column (1.0 x 50 mm, 1.7 μm). The composition of mobile phase A was 0.1% formic acid in water. The composition of mobile phase B was 0.1% formic acid in 3:1 acetonitrile: isopropyl alcohol. Gradient elution was employed, with mobile phase B being ramped from 20% at 0 to minutes, to 98% at 2 minutes and held for 2.5 minutes. The total run time was 5 minutes. Electrospray conditions for the μLC-ESI-MS/MS method were optimized for a flow rate of 65 μL/min and were as follows: Ion spray voltage was set to 5500 V, temperature of 150°C, curtain gas of 20, CAD gas of 10, and ion source gas 1 and 2 of 20 and 60 psi, respectively. Q1 and Q3 transitions for each analyte and voltage settings (*e*.*g*. collision energy, declustering potential) were optimized for each compound using a combination of manual tuning and compound optimization.

Concentrations of d_4_-α-tocopherol and d_4_-α-tocopheryl quinone in tissue homogenates were determined with μg analyte / g input tissue units, then converted to μM units using the appropriate analyte molecular weights and assuming the following tissue densities (liver 1.06 g/mL, heart 1.045 g/mL, small intestine 1.05 g/mL–refer to Appendix A of *Basics of Biomedical Ultrasound for Engineers*, H. Azhari, 2010). Quinone conversion percentages were calculated using the formula Q / (Q + C) *100 where Q = quinone concentration (in μM) and C = parent chroman concentration (in μM).

### ORAC assay

ORAC assay kits were purchased from Cell Biolabs. Assays were performed per the manufacturer’s instructions for lipophilic samples with minor modifications. DMSO stock solutions of compounds were diluted in an anaerobic glovebox to final concentrations of 25 and 12.5 μM in triplicate. A standard curve of Trolox^®^ between 0–100 μM in triplicate per concentration was also generated on each assay plate. Diluted compounds and standards were removed from the glovebox and reactions initiated by addition of aerobic fluorescein reagent and radical initiator. Fluorescein fluorescence was monitored at excitation wavelength of 480 nm and emission wavelength of 520 nm every minute for one hour. AUC analysis was performed as described in the kit protocol. The “Trolox equivalents” at each concentration of test compound were calculated and the average Trolox equivalents at the two compound concentrations are presented. The errors were determined by propagating errors from the SEMs of the triplicate determinations to the averages across the two triplicate determinations.

### Radical-trapping antioxidant (RTA) activity assays

Liposomes containing 2 mol% of **αT**, **αTCC**, **αTQ** or **αTHQ** in DOPC (18:1 1,2-dioleoyl-sn-glycero-3-phosphocholine) were prepared by thin-film hydration followed by extrusion to obtain large unilamellar vesicles at 120 nm in diameter with a polydispersity index (PDI) < 0.1 in DPBS buffer (pH 7.5). PDI measurements were determined using a Malvern Zetasizer Nano-ZSP.

RTA assays were prepared by diluting liposomes (to 2.5 mM [lipid]) in DPBS buffer and incubating at room temperature with 500 nM BODIPY™ 581/591 C11 dye for 15 minutes. A fresh stock of 20 mM AMVN (2,2’-azobis(4-methoxy-2,4-dimethylvaleronitrile), Wako Chemicals USA, Inc.) was prepared in MeOH and added to the liposome mix to a final concentration of 2 mM immediately before data collection. The decrease in the reduced dye’s fluorescence emission was measured every 30 seconds at 37°C, using excitation/emission of 575/600 nm with a 590nm cutoff filter, on a Molecular Devices Spectramax M2. The data plots were normalized to 100% fluorescence using the initial time points. The AUC analysis was performed using GraphPad Prism 7.

### Enzyme assay materials

Rabbit 15-lipoxygenase (rLOX15, part number BML-PL015, lot number 3-P9009a) was obtained from Enzo Life Sciences, Inc. Sodium cholate (part number C6445, lot number 094K0209) was obtained from Sigma-Aldrich. Dihydrorhodamine (part number 10055, lot number 12D0215) was obtained from Biotium. Arachidonic acid (part number 90010, lot number 0509246–4) and soybean 15-lipoxygenase (SLO, part number 60700, lot number 0500889–1) were obtained from Cayman Chemical. The 96 half-well black, flat bottom assay plates (part number 3993, lot number 19316045) were obtained from Corning, Inc.

### Lipoxygenase enzyme inhibition assay

Enzyme assays (100 μL final volume) were conducted in Corning 96 half-well black, flat bottom assay plates and contained final concentrations of 50 μM arachidonic acid as a substrate, 1:10 (v:v) cholate mix (2% (w:v) sodium cholate and 2% (v:v) DMSO), 40 μM dihydrorhodamine 123 and 3 U/mL rLOX15 in buffer A (100 mM Tris-HCl, pH 7.5). Stock solutions of test compounds were prepared in DMSO and then diluted to final assay concentrations in cholate mix such that final cholate and DMSO concentrations were maintained at 0.2%. Reactions were initiated by addition of 50 μL of enzyme mix (80 μM dihydrorhodamine 123 and 6 U/mL rLOX15 in buffer A) to wells containing 50 μL substrate and cholate mix in Buffer A and linear rates were assessed via fluorescence, using excitation/emission of 500/536 nm, on a Molecular Devices Spectramax M2 every 10 seconds for 5 minutes at room temperature.

### EPR sample preparation

Lipoxygenase: Soybean 15-lipoxygenase (SLO) was buffer exchanged into 50 mM borate buffer, pH 9.0 containing 10% (v/v) glycerol. The resulting protein solution was concentrated to approximately 100 μM, based upon the supplier’s activity per mL and specific activity per mg. The active-site iron oxidation state was further evaluated by EPR under three treatment conditions: 1) No treatment, 2) oxidation with 3-fold molar excess of lipid hydroperoxide (final concentration of 300 μM) from an ethanol stock, 3) oxidation with 3-fold molar excess of lipid hydroperoxide followed by 10-fold molar excess of **αTHQ** or **αT3HQ** (final concentration of 1 mM) from a DMSO stock. All samples contained the same final concentrations of ethanol and DMSO as sample 3. The EPR spectra of buffer alone were also evaluated to allow for baseline subtraction.

### Electron Paramagnetic Resonance (EPR) spectroscopy

#### Lipoxygenase

CW-EPR spectra were recorded at 150 K on a Bruker ELEXYS E540 spectrometer fitted with an X-band microwave bridge, a Super High Sensitivity Resonator (SHQ), and a Nitrogen VT apparatus. The spectra were recorded at 9.43 GHz with 1.002 mW microwave power, 10 G modulation amplitude, 100 kHz modulation frequency, 25 scans.

#### αTCC

CW-EPR spectra were recorded at room temperature in ambient air on a Bruker EMXnano spectrometer fitted with an X-band microwave bridge. All spectra were recorded at 9.61 GHz with 10 mW microwave power and 5 G modulation amplitude, 100 kHz modulation frequency, 25 scans.

### Targeted arachidonate metabolite release cellular assay

Ferroptosis was initiated by RSL3 (2 μM final concentration) in Q7 cells as described above. The protective activity of co-treated compounds was assessed also as described above. Four to five hours after RSL3 treatment, the conditioned medium was collected into 1.2-mL cluster tubes containing ice-cold acetonitrile at a ratio of 1:2. Tubes were snap frozen on dry ice and stored at -80°C.

Prior to analysis, samples were thawed on wet ice and then vortexed briefly. Samples were centrifuged at 4000 rpm for 15min at 4°C. Supernatant (40 μL) was transferred to a 96-well injection plate and 10 μL of both internal standard and HPLC grade water were added. A Shimadzu Nexera MP system coupled to a Sciex 6500 Qtrap and configured for micro-flow was used to analyze all samples. Samples were maintained at 4°C in the Nexera MP autosampler for the duration of the analytical run.

A Thermo Gold-C_8_ column (1.9 μm, 1.0 x 100 mm) operated at 45°C was used for chromatographic separation. The total run time of the liquid chromatography method was 10 min. Mobile phase A consisted of water with 0.1% formic acid. Mobile phase B was composed of 0.1% formic acid in 3:1 acetonitrile:isopropyl alcohol. Gradient elution was employed, with mobile phase B being ramped from 5% at 0min, to 50% at 2 min, and finally 98% at 7.5 min. Electrospray conditions for the μLC-ESI-MS/MS method were optimized for a flow rate of 125 μL/min and were as follows: Ion spray voltage was set to -4500 V, temperature of 250°C, curtain gas of 20, CAD gas of 10, and ion source gas 1 and 2 of 20 and 40 psi, respectively. Analyzer parameters were optimized for each compound using a combination of manual tuning and compound optimization. The following MRM transitions were used to quantify concentrations of 15-HETE, 12-HETE and Arachidonic Acid (AA). 15-HETE: 319→219; 12-HETE: 319→179; AA: 303→259.

### Statistical methods

All statistical analyses were performed using GraphPad Prism v.7.03 for Windows. If all groups showed apparently normal distributions (by Shapiro-Wilk test), then one-way ANOVA tests were performed, with Sidak’s correction for multiple comparisons applied when appropriate. If even one group did not meet normality assumptions, then a non-parametric Kruskal-Wallis test was used, with Dunn’s multiple comparisons test applied when appropriate. When only comparing two groups, then an unpaired *t*-test with Welch’s correction was used. Statistical significance throughout was defined as *p*<0.05.

## Results

**Fig 1:**
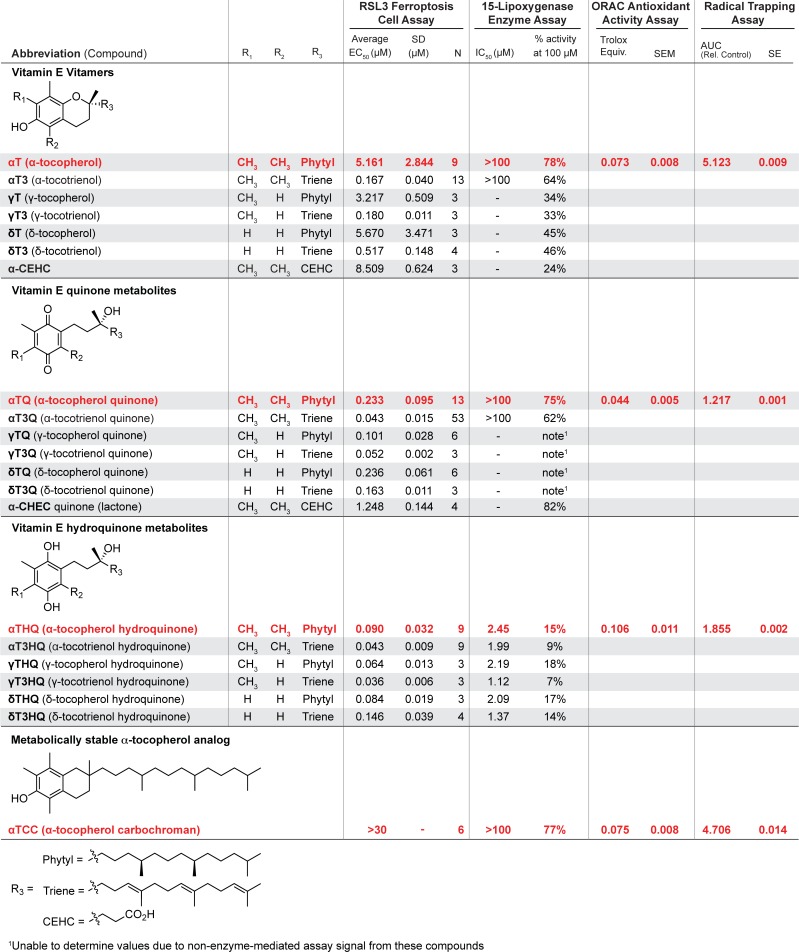
Ferroptosis cell rescue activity, 15-lipoxygenase enzyme assay data, ORAC and RTA values for 18 vitamin E vitamers and metabolites. Summary of cellular and enzyme assay potencies. Q7 ferroptosis cell rescue, 15-lipoxygenase (15-LO) enzyme inhibition, oxygen radical absorbance capacity (ORAC) values and the radical-trapping antioxidant (RTA) activity assays were measured for **αT**, its selected metabolites, and the non-metabolizable **αTCC**.

Previously, we reported that quinone metabolites of vitamin E have potent rescue activity in a glutathione (GSH) depletion cell survival assay which exhibited features of ferroptotic cell death [10–14]. To test the hypothesis that the quinone and/or hydroquinone metabolites of alpha-tocopherol (**αT**) are responsible for its previously reported anti-ferroptotic activity [6,15–19], we evaluated the activity of alpha-tocopherol quinone (**αTQ**) and hydroquinone (**αTHQ**) in a ferroptotic cell death assay using immortalized mouse striatal cells (ST*Hdh*^Q7/Q7^, hereafter referred to as Q7) [20]. These specific compounds were part of a larger screen which included 6 vitamin E vitamers and 14 known vitamin E metabolites (Fig 1) [21–23].]

**Fig 2:**
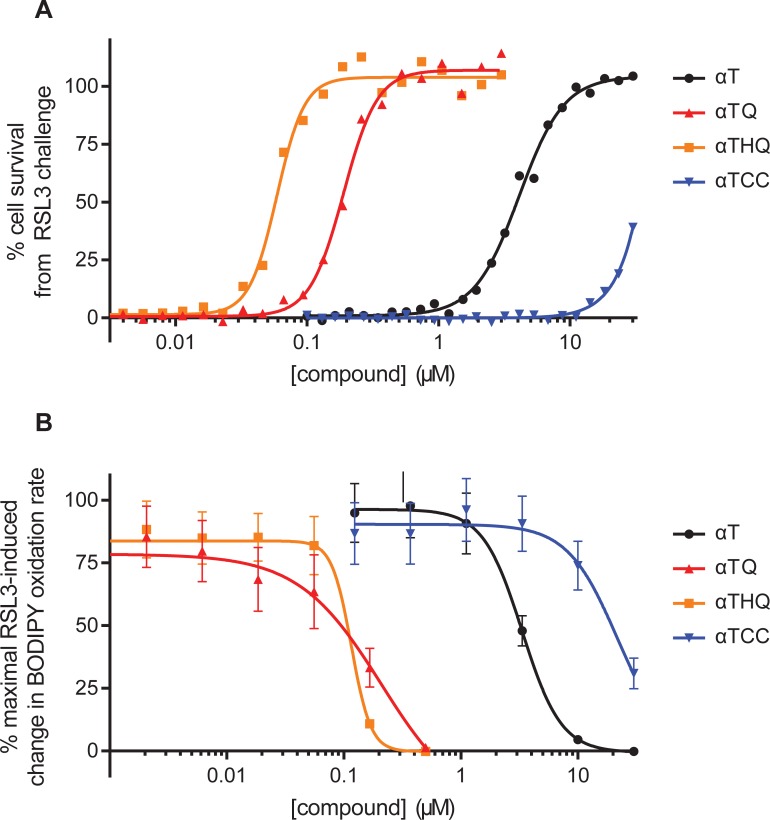
The anti-ferroptotic activity of vitamin E and its metabolites. (A) Ferroptosis was induced in Q7 cells by RSL3 treatment (2 μM 18 h), as measured by CellTiter-Glo^®^ 2.0 ATP assay. The dose-dependent cytoprotective activity of vitamin E (**αT**), its metabolites (**αTQ**, **αTHQ**), or the synthetic carbochroman (**αTCC**) was assessed by co-treatment (in 24-point titration) with RSL3. Curves are representative of ≥9 independent experiments, of which 7 evaluated **αTCC** to a top concentration of 30 μM. (B) Ferroptosis was initiated in BODIPY^TM^ 581/591 C11-prelabeled Q7 cells by RSL3 (2 μM) treatment, with cellular lipid oxidation assessed by the rate of change in green cellular fluorescence using time-lapse microscopy. Rescue compounds were co-treated (in 6-point titration) with RSL3. Mean ± SEM shown, (n = 6 to 9 technical replicates for each condition. Results are representative of 2 independent experiments.

As ferroptotic cell death has been shown to arise from genetic knockdown or chemical inhibition of phospholipid hydroperoxide glutathione peroxidase (GPX4) [6,9,24–26], we utilized (1*S*,3*R*)-RSL3, a well-described irreversible inhibitor of GPX4, to induce ferroptosis in Q7 cells. RSL3 treatment of Q7 cells resulted in dose- and time-dependent loss of cell viability. Typically, at RSL3 concentrations ≥1 μM, 80–95% loss of cell viability was observed 18 h after RSL3 treatment, with onset of cell death observed ~5 h after RSL3 addition. Consistent with published reports, **αT** prevented RSL3-induced cell death with an EC_50_ of 5.2 ± 2.8 μM (mean ± SD, N = 9; Fig 2). **αTQ** and **αTHQ** also prevented ferroptotic cell death, but were markedly more potent (22- and 57-fold, respectively) than **αT** itself (**αTQ** EC_50_ 0.23 ± 0.10 μM, N = 13; **αTHQ** EC_50_ 0.09 ± 0.03 μM, N = 9, Fig 2A). **αT**, **αTQ**, and **αTHQ** also prevented RSL3-induced lipid oxidation dose-dependently, following the same trend as their cytoprotective potency (as assessed by the prevention of BODIPY™ 581/591 C11 oxidation by time-lapse quantitative video microscopy, Fig 2B).

**Fig 3:**
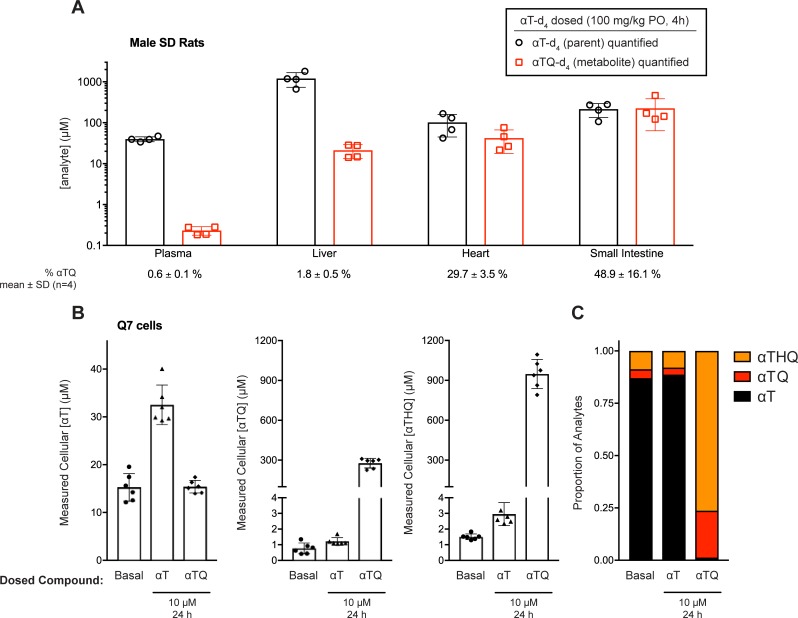
*In vivo* conversion of α-tocopherol to its quinone metabolite and quantification of intracellular concentrations of vitamin E and its metabolites in cells *in vitro*. (A) To assess the *in vivo* conversion of **αT** to its quinone metabolite, stable deuterium-labelled α-tocopherol (d_4_-**αT**) was dosed orally to Sprague-Dawley male rats. Four hours after the final dose, plasma and tissues were collected for bioanalytical quantification of d_4_-**αT** and d_4_-**αTQ** by LC-MS/MS. While <1% of deuterium-labeled **αT** was detected as the quinone in the plasma fraction, varying levels of quinone conversion were observed in the tissues assessed, ranging from ~2% (liver) to ~50% (small intestine). (B) Vitamin E (**αT**) and its metabolites, vitamin E quinone (**αTQ**) and vitamin E hydroquinone (**αTHQ**), were detected simultaneously in Q7 striatal cells under basal growth conditions or supplementation with **αT** or **αTQ** (10 μM, 24 h). Results displayed are mean ± SD, n = 6; results from 1 experiment representative of 3 similar experiments. (C) Stacked bar graphs showing the mean proportions of **αT**, **αTQ**, and **αTHQ** quantified simultaneously under basal, **αT**- or **αTQ**-supplemented conditions using the succinate capping methodology described in Methods.

The improved anti-ferroptosis potency of the **αTQ** and **αTHQ** metabolites compared to the parent **αT** led us to assess the prevalence of conversion in biological systems. To demonstrate *in vivo* the oxidative chroman ring opening of **αT** to **αTQ**, we synthesized stable-labelled d_4_-**αT** and dosed fed male rats once daily for four days by oral gavage at 100 mg/kg (S1 Method). The tissues and plasma were isolated four hours after the final dose and analyzed by LC-MS/MS to determine the concentrations of both d_4_-**αT** and d_4_-**αTQ**. The conversion and distribution of d_4_-**αT** and d_4_-**αTQ** was tissue- and compartment-specific, ranging from a ratio of 1:1 (d_4_-**αT**:d_4_-**αTQ**) in the small intestine, reaching concentrations of approximately 200 μM, to plasma where the (d_4_-**αT**:d_4_-**αTQ**) ratio was greater than 100:1. Due to the rapid and spontaneous re-oxidation of the hydroquinone (**αTHQ**) to the quinone (**αTQ**) [27], we did not attempt to quantify the tissue **αTHQ** exposure in this study. In summary, these results directly demonstrate the *in vivo* conversion of **αT** to **αTQ** and are consistent with the findings of others (Fig 3A)[28–36].

To assess directly the cellular exposure of **αT** and its metabolites, **αTQ** and **αTHQ**, in our cellular ferroptosis model system, we established an LC-MS/MS method to quantitate all three analytes simultaneously in cell extracts. Because **αTHQ** is rapidly oxidized back to **αTQ** upon exposure to atmospheric oxygen, [27] we quantified its cellular concentration by trapping it as the stable *bis*-succinate ester [37]. We observed that Q7 striatal cells cultured under basal growth conditions had detectable **αT**, **αTQ**, and **αTHQ** (**αT**:**αTQ**:**αTHQ** proportions 88:3:8; Fig 3B and 3C), likely due to the **αT** (quantified at 2.3 μM) in the culture medium containing 10% fetal bovine serum. After **αT** supplementation (10 μM, 24 h) in the Q7 cell medium, the cellular **αT**, **αTQ**, and **αTHQ** concentrations were each elevated (Fig 3B), yet maintained a similar proportional distribution (**αT**:**αTQ**:**αTHQ**, 87:4:9; Fig 3C) to those observed under basal growth conditions. These observations suggest that a relationship exists between the concentration of **αT** and the corresponding concentrations of **αTQ** and **αTHQ**, likely mediated by **αT** to **αTQ** oxidative conversion and subsequent reduction to **αTHQ** through a cellular activity such as the widely-expressed NAD(P)H:quinone oxidoreductase 1 (NQO1) [38,39]. When Q7 cells were supplemented with **αTQ** (10 μM, 24 h), the cellular exposure of **αT** was unaffected (Fig 3B, left panel), demonstrating that the dosed **αTQ** did not biochemically “back-convert” to **αT** in these cells. By contrast, supplementation with **αTQ** led to a dramatic increase in cellular **αTQ** and **αTHQ** exposure (**αTQ**, >300-fold increase, to over 250 μM; **αTHQ**, >600-fold increase, to over 900 μM; Fig 3B). **αTQ** supplementation of Q7 cells resulted in intracellular **αT**:**αTQ**:**αTHQ** exposure ratios of 1:22:76 (Fig 3C), a marked alteration from the baseline proportions. These results demonstrate that the dosed quinone (**αTQ**) is reduced to the hydroquinone (**αTHQ**) form readily in cells; indeed, the **αTHQ** exposures exceed those of **αTQ** by 3- to 4-fold in all cases tested.

**Fig 4:**
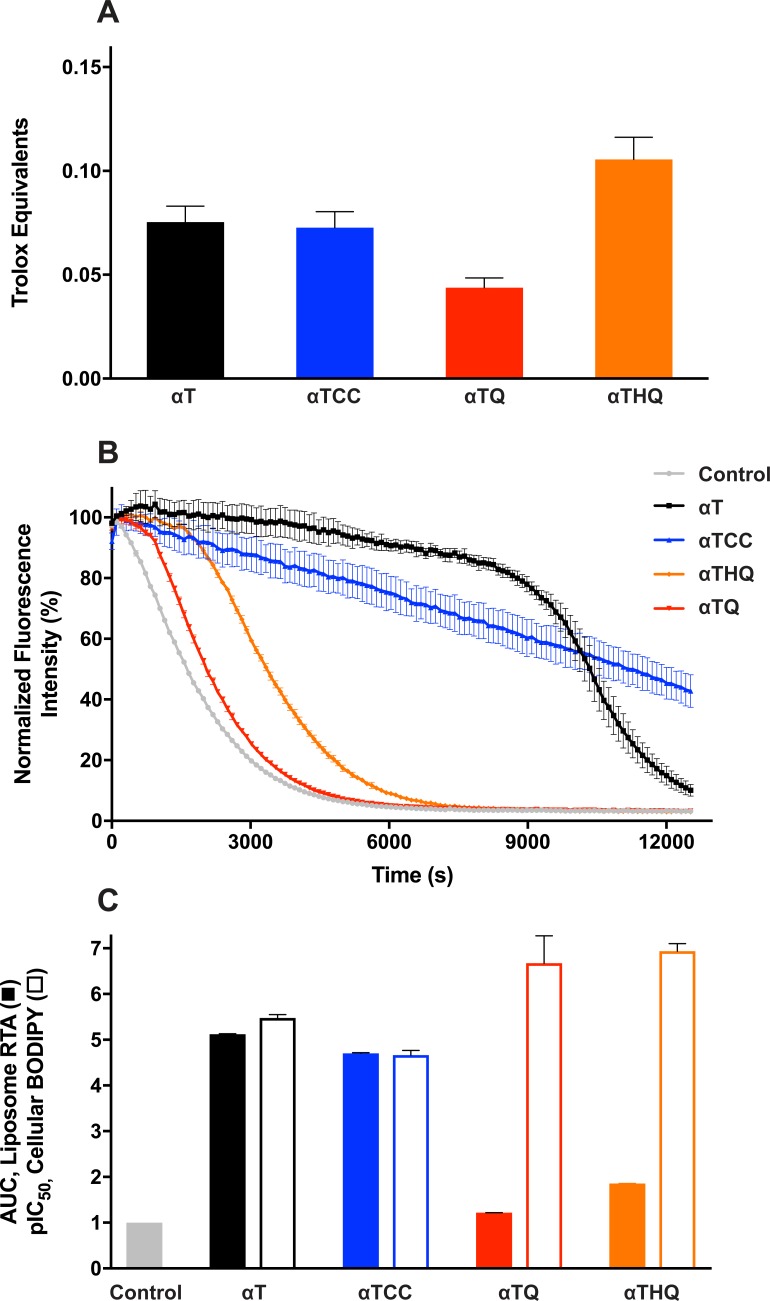
Oxygen radical absorbance capacity (ORAC) and radical-trapping antioxidant (RTA) activity assays with αT, αTCC, αTQ, αTHQ in comparison with their ferroptosis rescue potency. (A) ORAC activity of each compound compared to Trolox. Mean ± SEM (N = 6) displayed. (B) Overlay of kinetics of RTA activity of each compound (50 μM) prepared as 2 mol% in DOPC liposomes, detected through quenching of BODIPY™ 581/591 C11 (0.5 μM) emission at 600 nm. Mean ± SD (N = 3) displayed. (C) Comparison of RTA BODIPY quenching area-under-the-curve (AUC) of each compound (relative to the Control group, defined as 1) and pIC_50_ values for inhibition of RSL3-induced cellular BODIPY oxidation. Mean + SEM displayed; N = 3 for RTA assay, N = 6 for cell BODIPY assay.

To test whether metabolic conversion of the **αT** chroman ring to the corresponding **αTQ** quinone is necessary for anti-ferroptotic activity, we designed and synthesized a novel non-metabolizable analog of **αT** we refer to as **αT** carbochroman (**αTCC**, Fig 1). **αTCC** is a conservative, one-atom, oxygen-to-methylene substituted analog of **αT** which retains the same phenolic group in **αT** that is widely regarded as the moiety responsible for vitamin E’s radical scavenging activity [40] (see S2 Method. Procedure for the synthesis of **αTCC**). We confirmed that, similar to **αT**, **αTCC** forms a radical through hydrogen atom donation, and this radical is delocalized over the carbochroman ring system (S1 Fig). Furthermore, when assessed in a solution-based oxygen radical absorbance capacity (ORAC) assay, as expected, **αTCC** had comparable antioxidant activity to **αT** (0.075 ± 0.008 vs. 0.073 ± 0.008 Trolox equivalence units, respectively; mean ± SEM, n = 6 each; p>0.05 by two-tailed t-test; Figs 1 and 4A) [41]. Despite the similarity in antioxidant activity and radical formation, however, the anti-ferroptotic cellular activity of **αTCC** was markedly decreased compared to **αT**, with an EC_50_ > 30 μM (Fig 2A) and only 42 ± 21% (mean ± SD; N = 7 independent experiments) rescue at 30 μM, the highest concentration tested due to solubility limitations. We confirmed cellular exposure of **αTCC** to be >50 μM (6 technical replicates in each of 4 independent experiments, S3 Method), indicating that the weak rescue activity was not due to poor cellular uptake.

Ferroptosis occurs via oxidation of the lipid membrane, the primary location of the highly lipophilic vitamin E species. We next sought to determine whether the rank order of antiferroptotic potency of **αT**, **αTQ**, **αTHQ**, and **αTCC** could be predicted by antioxidant activity in a more biologically relevant lipid environment. We therefore tested the radical-trapping activity (RTA) of **αT**, **αTCC**, **αTQ**, and **αTHQ** in liposome vesicles as measured by an increased lag period prior to quenching of BODIPY™ 581/591 C11 fluorescence (Fig 4A) [8,42]. Under these conditions we found that the time to quench BODIPY™ 581/591 C11 fluorescence in DOPC liposomes showed a rank order of **αT**> **αTCC**>> **αTHQ**> **αTQ** (Fig 4B). This result is further highlighted by an area-under-the-curve (AUC) comparison of each compound determined with respect to the liposome only control, which shows that both **αT** and **αTCC** have similar activity and are between 2.5–4 fold greater than **αTQ** and **αTHQ** (Fig 4B). These data are in stark contrast to the concentrations required to prevent RSL3-induced cellular BODIPY™ 581/591 C11 oxidation (Fig 4B, compare open *vs*. closed bars), and argues that radical trapping antioxidant activity alone is not sufficient to explain the increased potency of **αTQ** and **αTHQ** (Fig 4C). Our results suggest that the radical scavenging activity of **αT** is not sufficient to prevent ferroptosis, consistent with the hypothesis that the quinone/hydroquinone metabolites are largely responsible for the anti-ferroptotic effect attributed to **αT**. Furthermore, while **αTHQ** exhibits weak but quantifiable ORAC and RTA activity, these activities alone cannot explain the anti-ferroptotic potency of **αTHQ** (Fig 4B and 4C).

**Fig 5:**
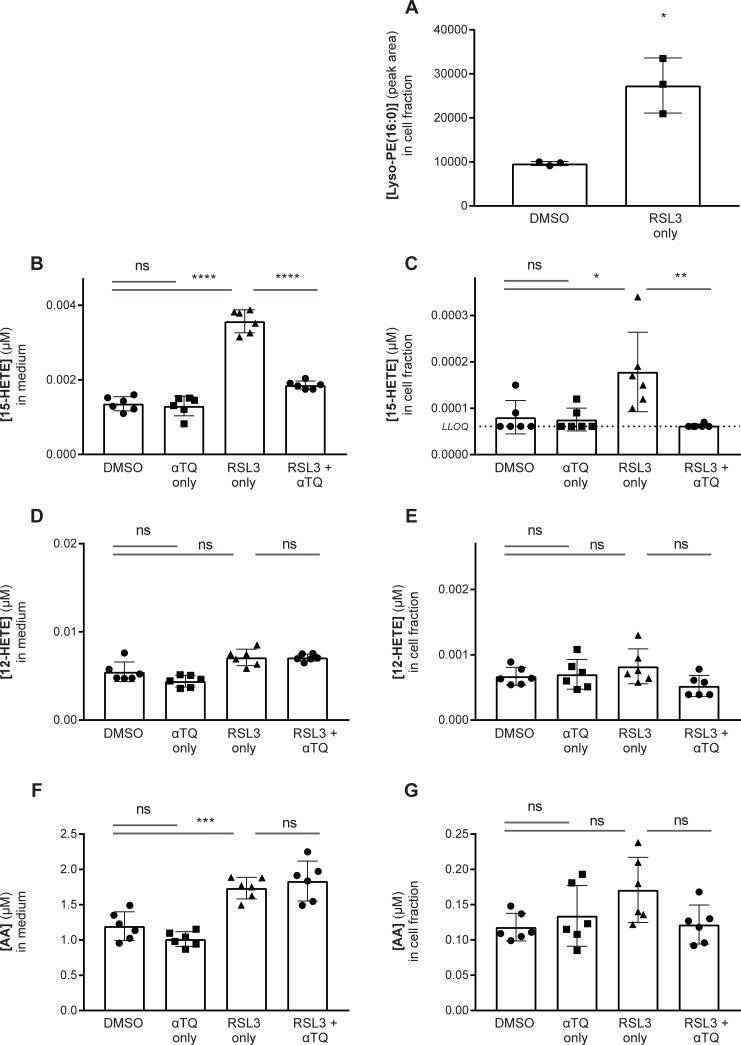
Lipid changes quantitated in RSL3-treated Q7 cells or their culture medium. (A) RSL3-Q7 treated cells have elevated cellular levels of a lyso-PE-palmitoyl species (*p<0.05, Unpaired t-test with Welch’s correction). (B, C) RSL3-treated Q7 cells have elevated cellular and conditioned media levels of 15-HETE, an effect prevented by **αTQ** (5 μM) co-treatment (*p<0.05, **p<0.01, ***p<0.001, Shapiro-Welk normality test followed by ANOVA with Sidak’s multiple comparisons test or Kruskal-Wallis test with Dunn’s multiple comparisons test, respectively). (D, E) 12-HETE levels were not altered by RSL3 or **αTQ** treatment (ns, p>0.05, Shapiro-Welk normality test followed by Kruskal-Wallis test with multiple comparisons test. (F, G) Conditioned medium of RSL3-treated Q7 cells has elevated AA, but cellular levels are not significantly changed (***p<0.001, Shapiro-Welk normality test followed by ANOVA with Sidak’s multiple comparisons test).

It has been reported that ferroptosis requires lipoxygenase-mediated production of peroxidated polyunsaturated fatty acids [6,26]. Therefore, we studied the ability of the **αT** metabolite, **αTQ**, to modulate the generation of endogenous oxidized lipids in Q7 cells [38]. During untargeted LC-MS/MS analysis of Q7 cells undergoing RSL3-induced ferroptosis, we observed a significant increase in a lyso-phosphatidylethanolamine(PE)-palmitoyl species (*m/z* = 436.284, neg. mode), suggesting that free fatty acids might be released during this death process (Fig 5A). Given the central role of arachidonate-containing PE lipids in ferroptosis [2,6,26,43], we assessed whether arachidonic acid (AA) and/or 19 of its known metabolites were released from cells into the culture medium during RSL3 treatment (S1 Table). Interestingly, in three independent experiments, only two analytes were significantly altered by RSL3 treatment: 15-hydroxyeicosatetraenoic acid (15-HETE) and AA itself (p<0.05 by Kruskal-Wallis test with Dunn’s correction for multiple comparisons, Fig 5B and 5F). None of the hydroperoxidated arachidonyl species were detected in our experiments. We also observed that cellular free 15-HETE but not AA was induced by RSL3 treatment (Fig 5C and 5G). Importantly, the RSL3-dependent induction of both cellular and released 15-HETE were inhibited by **αTQ** (5 μM, Fig 5B and 5C); by contrast, **αTQ** treatment had no effect on cellular or released AA levels (Fig 5F and 5G). Finally, we note that neither cellular nor released 12-HETE levels were induced by RSL3 treatment, suggesting a specific role for 15-lipoxygenase in Q7 cells undergoing ferroptosis (Fig 5D and 5E).

**Fig 6:**
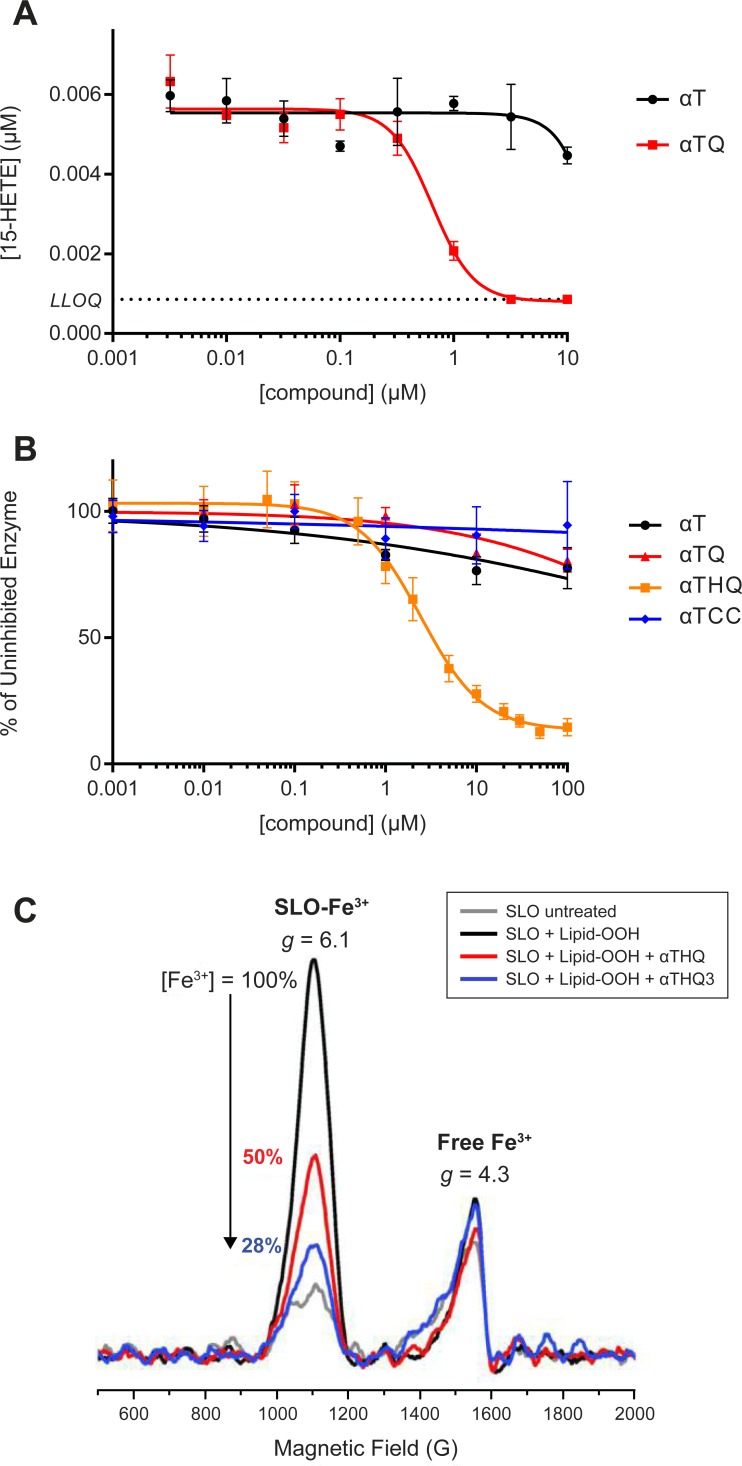
Inhibitory activity of 15-LO by vitamin E and its metabolites in an isolated enzyme assay and in Q7 cells. (A) **αTQ** dose-dependently prevents RSL3-induced cellular release of 15-HETE (IC_50_ ~0.6 μM). Mean ± SD shown (n = 3), representative of 2 independent experiments. (B) **αTHQ** inhibited rabbit 15-lipoxygenase (15-LO) enzyme activity (IC_50_ 2.45 μM) utilizing arachidonic acid as substrate, whereas **αT**, **αTQ**, and **αTCC** did not (up to 100 μM). Data are mean ± SD (n = 4, from 2 independent experiments). (C) EPR spectroscopy of soybean 15-lipoxygenase (SLO) with **αTHQ** or **αT3HQ**. Overlaid spectra focusing on the active site Fe^3+^ (g = 6.1) and the exogenous rhombic Fe^3+^ that is typically present in purified protein fractions (g = 4.3). Quantification of the Fe^3+^-SLO before treatment, after oxidation by lipid-hydroperoxide (set to 100%), and after compound treatment.

We sought to determine the inhibitory potency of the parent **αT** or its quinone metabolite **αTQ** on RSL3-induced cellular 15-HETE release. When Q7 cells were cotreated with RSL3 and **αT,** 15-HETE production was reduced by only 19% at 10 μM with **αT**. In contrast, **αTQ** dose-dependently returned 15-HETE concentration to background levels (IC_50_ = 0.6 μM; Fig 6A) with a potency similar to its cytoprotective activity (EC_50_ = 0.23 μM; Figs 1 and 2A). The dose-dependent inhibition of 15-LO mediated lipid metabolites by **αTQ** at similar concentrations to its cellular antiferroptotic potency suggests that these two activities might be mechanistically linked.

To determine whether the superior anti-ferroptotic potency of **αTHQ** and **αTQ** relative to **αT** and **αTCC** could be due to direct inhibition of 15-LO enzyme activity, the rate of AA peroxidation using purified rabbit 15-LO was measured over a range of compound concentrations. Only **αTHQ** displayed potent inhibition of 15-LO (IC_50_ = 2.45 μM), while **αT**, **αTCC**, and **αTQ** all failed to decrease enzyme activity by more than 20% at 100 μM, the highest concentration tested (Fig 6B). These data demonstrate that the 15-LO inhibitory potency of **αTHQ** is in excess of 40-fold when compared with the potency of either **αTQ**, **αT**, or **αTCC**.

The catalytic activity of 15-Lipoxygenase requires a non-heme iron center to be in the Fe^3+^ state. We investigated whether the inhibitory activity of **αTHQ** resulted from the reduction of the 15-LO Fe^3+^ to its inactive Fe^2+^ state using the 15-LO enzyme from soybean (SLO), and EPR spectroscopy to measure its redox state [44,45]. Incubation of SLO with α-linoleic acid hydroperoxide (lipid-OOH) converted all of the iron to the Fe^3+^ state, which was spin quantified and set to 100% Fe^3+^ (Fig 6C). Upon addition of **αTHQ**, a 50% decrease in the SLO-Fe^3+^ signal was observed. A lack of change in the exogenous rhombic Fe^3+^ at *g* = 4.3 (which is not bound to SLO) indicated that Fe^3+^ reduction by **αTHQ** was specific to the enzyme-bound metal.

To determine the key molecular features of vitamin E and its metabolites that afford the ability to inhibit 15-LO and ferroptosis, we prepared a library of each of the naturally occurring vitamers (α-, γ- and δ-tocopherols and tocotrienols) and their major metabolites (Fig 1). With this library, we related structural changes in the chroman head, lipid tail, and quinone ring functionality with 15-LO inhibition and protective anti-ferroptotic activity. Of particular interest was determining whether the two major forms of vitamin E, tocopherols and tocotrienols, function by the same mechanism-of-action. Consistent with Kagan *et al*., [6] we observed that **αT3** was more potent in inhibiting ferroptosis than **αT** (~30-fold, Fig 1), a trend that was also observed for the γ-, and δ-forms (~18- and ~10-fold, respectively). We also found that the quinone and hydroquinone metabolites of α-, γ- and δ-tocotrienols were more potent than their parent chroman forms. This anti-ferroptotic activity was due to a similar mechanism of action as the tocopherol series: **αT3HQ** also potently inhibited 15-LO enzymatic activity and reduced the Fe^3+^ catalytic center in SLO (Figs 1 and 6C). The potent anti-ferroptotic activity (EC_50_ = 0.043 μM) of alpha-tocotrienol quinone **αT3Q**, together with a favorable pharmacokinetic and safety profile, position it as an attractive candidate to treat diseases involving oxidative stress, inflammation, and ferroptosis[14].

## Discussion

**Fig 7:**
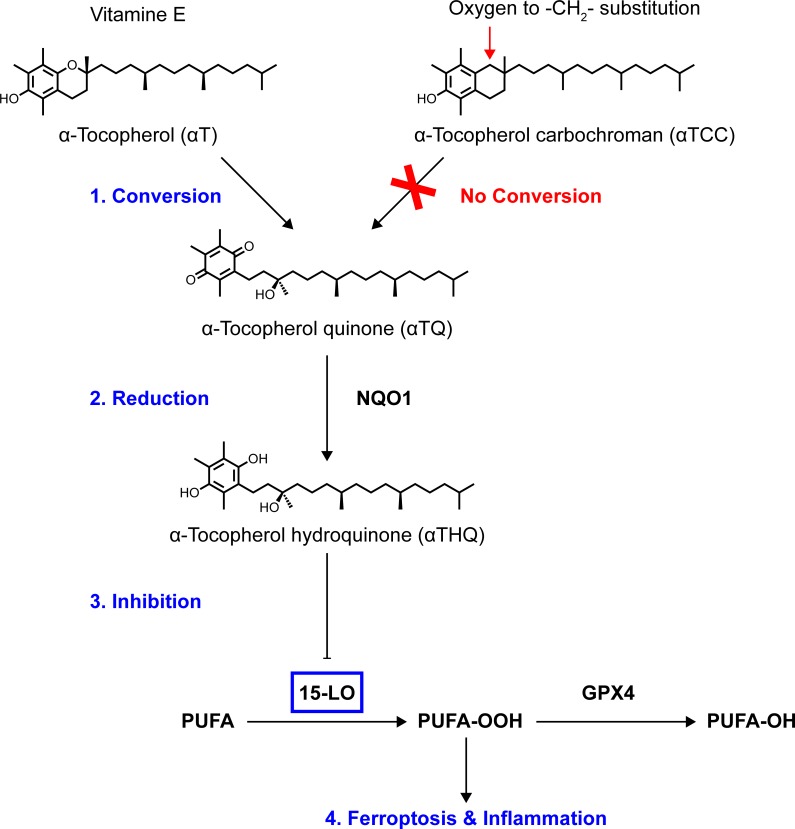
Proposed target and mechanism of action of vitamin E. The anti-ferroptotic effect of vitamin E results from a four-step biochemical mechanism: 1) oxidative hydrolysis of alpha-tocopherol (**αT**) to alpha-tocopherol quinone (**αTQ)**; 2) reduction of alpha-tocopherol quinone (**αTQ**) to alpha-tocopherol hydroquinone (**αTHQ**); 3) inhibition of 15-LO via reduction of its non-heme Fe^3+^ center to the inactive Fe^2+^ state by alpha-tocopherol hydroquinone (**αTHQ**); and 4) inhibition of the ferroptosis cascade by blocking formation of lipid peroxidation products: polyunsaturated fatty acids (PUFA), hydroperoxidated polyunsaturated fatty acid (PUFA-OOH) and hydroxylated polyunsaturated fatty acid (PUFA-OH). The non-metabolizable isosteric analog of vitamin E **αTCC** retains its antioxidant activity, but does not inhibit ferroptosis.

In summary, we dissected the structural and enzymatic basis for vitamin E pharmacology based on protection from ferroptosis. To address the prevailing hypothesis that the lipid antioxidant activity of vitamin E (**αT**) is responsible for its anti-ferroptotic activity, we used the novel, non-metabolizable **αT** analog, **αTCC**, as a chemical biology probe and found that the radical trapping activity of **αT** is insufficient to explain its anti-ferroptotic cellular activity (Fig 4C). We present evidence that the anti-ferroptotic biological activity of vitamin E results instead from a direct action of its hydroquinone metabolite on the lipoxygenase active site Fe^3+^ center (Fig 7). Specifically, using the data reported herein, we propose the following model for vitamin E mechanism-of-action: (1) oxidative chroman ring opening of **αT** to its quinone metabolite, **αTQ** (Fig 3A); (2) *in situ* reduction of **αTQ** to its hydroquinone form (**αTHQ**; Fig 3B and 3C); deactivation of lipoxygenase enzyme activity (Fig 6B) by **αTHQ** via direct reduction of the active-site Fe^3+^ to its inactive Fe^2+^ state (Fig 6C); (4) thereby preventing cellular lipid oxidation (Figs 2B, 4B, 5B, 5C and 6A) and subsequent ferroptotic cell death (Fig 2A).

In 1939, Evans and Bishop noted that both vitamin E (**αT**) and vitamin E quinone (**αTQ**) possessed protective activity in fetal resorption studies[46,47]. For reasons that were not articulated in their initial work, vitamin E (**αT** chroman) was defined as the essential nutritional factor, not the vitamin E quinone. More recently, a vitamin E effect on fetal resorption was demonstrated in mice by action at the 15-LO/GPX4 ferroptotic pathway[7,48,49]. Our experimental evidence suggests that the hallmark activity leading to the classification of alpha-tocopherol as a vitamin may be in fact mediated by its metabolite, vitamin E hydroquinone.

## Supporting information

S1 Method The synthesis of d_4_-α-tocopherol (d_4_-αT) and d_4_-α-tocopherol quinone (d_4_-αTQ). (DOCX)

S2 Method The synthesis of αTCC–alpha tocopherol carbochroman. (DOCX)

S3 Method Cellular quantification of alpha-tocopherol carbochroman (αTCC). (DOCX)

S1 Fig EPR spectrum of the radical formed upon oxidation of αTCC. (DOCX)

S1 Table The metabolites measured in culture medium during ferroptosis by LC-MS/MS. (DOCX)
