# Supplementary material for: Vitamin E hydroquinone is an endogenous regulator of ferroptosis via redox control of 15-lipoxygenase
Source: PLoS One. 2018 Aug 15;13(8):e0201369. doi: 10.1371/journal.pone.0201369 (PMC6093661; doi:10.1371/journal.pone.0201369)
Supplement: S1 Method — (DOCX) [file pone.0201369.s001.docx]

# **Supporting Information**

## **S1 Method. The synthesis of d_4_-α-tocopherol (d_4_-αT) and d_4_-α-tocopherol quinone (d_4_-αTQ)**

General Chemistry Procedures

The following abbreviations were used in this section:

AcOH – Acetic acid

DCM – Dichloromethane

DME – 1,2-dimethoxyethane

DMF – Dimethyl formamide

DMSO – Dimethyl sulfoxide

LAH – Lithium aluminum hydride

LDA – Lithium diisopropylamide

MeOH – Methanol

MOMCl – Methoxymethyl chloride

MsCl – Methanesulfonyl chloride

MTBE – Methyl *tert*-butyl ether

Pd/C – Palladium on activated carbon

TFA – Trifluoroacetic acid

TFAA - Trifluoroacetic anhydride

THF – Tetrahydrofuran

TLC – Thin layer chromatography

All reagents were obtained from commercial suppliers and used without further purification unless otherwise stated.

Synthesis of d_4_-α-Tocopherol and d_4_-α-Tocopherol Quinone from δ-Tocopherol, as adapted from US Patent 8,106,223, Wesson, K., Hinman, A., Jankowski, O. Jan 31, 2012.

**SCHEME 1**

**(*R*)-2,8-dimethyl-5,7-bis((4-methylpiperazin-1-yl)methyl-d_2_)-2-((4*R*,8*R*)-4,8,12-trimethyltridecyl)chroman-6-ol**

The reaction vessel was assembled as follows: a 250 mL, two necked, round bottom flask was equipped with a stir bar, a nitrogen inlet /outlet at port 1 (which doubles as an addition port), and a thermocouple via an adaptor at port 2. The vessel was then degassed with nitrogen gas for not less than 10 min. Chromatographically purified δ-Tocopherol (38 g, 9.45 mmol) was added rinsed with no more than 30 mL of Me-piperazine (3 portions). To δ-tocopherol was added deuterated paraformaldehyde ((CD_2_O)_n_, 8.8 g, 275 mmol) and rinsed with Me-piperazine (no more than 5 mL). The mixture was then sealed and stirred at ca 75 °C and 500 RPM for 3 h. Suspension appeared clear yellow/orange with white solids. After, 3h, the mixture was then stirred at ca 111 °C and 1500 RPM for 24 h.

At the end of 24 h, the mixture was allowed to cool to room temperature, stirring at 100 RPM. Suspension appeared clear dark orange. To the reaction mixture was charged acetonitrile (MeCN, 150 mL), and transferred to a 2L RB flask. The vessel was then rinsed with MeCN (no more than 2 x 150 mL) and the contents transferred to vessel Y. Vessel Y was then degassed with nitrogen and the contents stirred at 800 RPM. To the mixture was added heptane (450 mL). Then Formic Acid was added via a pressure equalizing dropping funnel (PEDF) (150 mL) at a rate of ca 5 ml/min. Gas evolution was observed. Very little to no exotherm was observed. After addition, the contents of vessel Y was transferred to a 2 L separating funnel, degassed and allowed to settle (no less than 5 min). The bottom MeCN layer appeared clear dark orange. The top heptane layer appeared clear light yellow.

The MeCN layer was transferred into vessel Z using no more than 5 mL of MeCN to rinse. Reaction vessel Z was assembled as follows: a 2 L, two necked, RB flask was equipped with a stir bar, a nitrogen inlet at port 1 (which doubles as an addition port) and a nitrogen outlet at port 2. A cold water bath was equipped to the bottom of vessel Z to prevent overheating. A solution of aqueous potassium phosphate (45% w/w K_3_PO_4_, 400 mL) was then added via a PEDF at ca 30 min/mL and stirred at 900 RPM. Very little exotherm was observed. After addition, the organic and aqueous layers were allowed to settle (no less than 5 min) and the lower aqueous layer tested for pH. pH should be between 8-10. The contents of vessel Z was then transferred into a 2 L separating funnel with no more than 150 mL of MTBE to rinse. The separating funnel was degassed and the two layers allowed to settle for no less than 5 min. The layers were then separated. The top organic layer appeared clear orange. The bottom aqueous layer appeared a little cloudy yellow. To the separating funnel was added brine solution (20% w/w NaCl, 150 mL) and the layers allowed to settle for no less than 5 min. The layers were then separated. The top organic layer appeared clear orange. The bottom layer appeared clear orange/brown.

The organic layer was then dried with sodium sulfate (Na_2_SO_4_, ca 20 g.) and concentration via rotary evaporation under vacuum at 40 °C and at 100 torr (MTBE), then 30 torr (MeCN), then 0 torr. Then the MeCN in the crude material was chased off with toluene (3 x 10 mL) until constant mass was obtained. Approximately 60 g of yellow/orange crude material was obtained.

**d_4_-α-Tocopherol**

**(*R*)-2,8-dimethyl-5,7-bis(methyl-d_2_)-2-((4*R*,8*R*)-4,8,12-trimethyltridecyl)chroman-6-ol**

Reaction vessel X was assembled as follows: a 1 L, three necked, round bottom flask was equipped with a rubber septum at port 1 (addition and sampling port), an overhead stirrer and nitrogen inlet at port 2 and a thermocouple via an adaptor at port 3 (which doubled as a nitrogen outlet). Vessel X was then degassed with nitrogen gas for not less than 10 min. Sodium cyanoborohydride (NaCNBH_3_, 35.8 g, 570 mmol) and 3-methyl-1-butanol (amyl-OH, 230 mL) was then charged to vessel X via port 1 and heated to 125 °C and stirred at 50 RPM for 1h. Then a prepared solution of crude concentrate from step 1 (60.0 g) in amyl-OH (100 mL) was charged to the preheated mixture in vessel X via syringe pump at ca 20 mL/hr. Evolution of gas (HCN) was observed. After addition, the mixture was continued to stir at 200 RPM and ca 130 °C for 18 h. The mixture appeared clear yellow.

At the end of 18 hrs, vessel X was removed from the heating source and cooled to ca 50 °C. Mixture appeared cloudy yellow/orange. Then heptanes (250 mL) was charged to the mixture and cooled to ca 0 °C and continued to stir for no less than 10 min. Then an aqueous solution of potassium phosphate (45% w/w K_3_PO_4_, 250 mL) was charged via a pressure equalizing dropping funnel (PEDF) at ca 5 ml/min and an internal temperature of no more than 5 °C. After addition, the mixture was stirred for and addition 1.5 hrs at room temperature and 300 RPM.

After stirring, the mixture was allowed to settle for no less than 5 min and then both layers were split. The top organic layer appeared clear orange. The bottom aqueous layer appeared cloudy pale white. The aqueous layer was then treated with glyoxal (40% wt, 30 mL) to neutralize residual HCN. The top organic layer appeared clear orange. The organic layer was then dried with sodium sulfate (Na_2_SO_4_, ca 10 g.) and concentration via rotary evaporation under vacuum at 60 °C and 0 torr. Then the crude material was chased with heptanes (3 x 10 mL) until constant mass was obtained. About 25 g of orange/brown crude material was obtained.

Isolation was performed via silica gel column chromatography, eluting from 1% to 9% Ethyl Acetate in Heptanes. About 14 g of clear colorless oil was obtained (ca 34% yield). Upon storage overnight in a freezer, the oil becomes a pale yellow/white solid.

^1^H-NMR (400MHz, CDCl_3_, 25 °C): δ = 4.14 (s, OH), 2.61 (t, 2H, *J* = 6.9 Hz), 2.16 (s, 1H), 2.11 (s, 3H), 2.08 (m, 1H), 1.79 (m, 2H), 1.53 (m, 3H), 1.27 (m, 18 H), 1.23 (s, 3H), 0.87 (m, 12 H). ^13^C-NMR (400MHz, CDCl_3_, 25 °C) δ 145.53, 144.54, 122.61, 120.90, 118.35, 117.35, 74.50, 39.81, 39.37, 37.47, 37.44, 37.42, 37.28, 32.79, 32.70, 31.54, 27.97, 24.79, 24.44, 23.79, 22.71, 22.68, 22.62, 21.03, 20.74, 19.74, 19.65, 14.10, 11.75.

**^1^H and ^13^C NMR spectra for d_4_-α-Tocopherol**

**d_4_-α-Tocopherol quinone (d_4_-αTQ)**

**(2-((3*R*,7*R*,11*R*)-3-hydroxy-3,7,11,15-tetramethylhexadecyl)-6-methyl-3,5-bis(methyl-d_2_)cyclohexa-2,5-diene-1,4-dione**

Reaction vessel X was assembled as follows: a 100 mL, three necked, round bottom flask was equipped with a thermocouple at port 1, a pressure equalizing dropping funnel (PDEF) and a nitrogen inlet at port 2, and a nitrogen outlet at port 3. Vessel X was then degassed with nitrogen gas for not less than 10 min. d_4_-α-Tocopherol (5.0 g, 11.5 mmol), isopropyl acetate (IPAc, 40 mL) and DI water (10 mL) was charged into vessel X at room temperature and stirred at 50 RPM. The mixture was then cooled to no more than 0 °C and stirred at 900 RPM.

In a 25 mL graduated cylinder, Cerium ammonium nitrate (CAN, 13.25 g, 24.4 mmol) was dissolved in DI water (10 mL). After addition, the mixture was stirred for no less than 30 more minutes to afford a yellow/orange solution. After stirring, the CAN solution was then added via a PEDF into vessel X over a period of 15-30 min while maintaining an internal temperature of no more than 5 °C. The suspension was stirred for an additional 30 min at 1500 RPM while maintaining an internal temperature of no more than 5 °C.

After 30 min, the suspension was allowed to settle and the two layers were split (split in less than 10 s) while still cold. The top organic layer appeared clear orange. The bottom aqueous layer appeared clear with a yellow tint. The organic layer was then concentrated *in vacuo* at no more than 35 °C and the residual solvent chased off with heptanes (3 x 10 mL) at 0 torr until constant mass we obtained. Approximately 5 g of orange oil was obtained. Purification and isolation was performed via silica gel chromatography, eluting from 3% to 10% Ethyl Acetate in Heptanes. Approximately 4.5 g of clear orange oil was obtained (ca 87% yield).

^1^H-NMR (400MHz, CDCl_3_, 25 °C): δ = 2.54 (t, 2H), 2.00 (s, 3H), 2.00 (m, 2H), 1.50 (m, 6H), 1.29 (m, 12H), 1.23 (s, 3H), 1.12 (m, 6H), 0.85, (m, 12H). ^13^C-NMR (400MHz, CDCl_3_, 25 °C) δ 187.69, 187.19, 144.46, 140.42, 140.06, 77.30, 76.98, 76.67, 72.63, 42.27, 40.26, 39.35, 37.59, 37.42, 37.26, 32.78, 32.75, 27.95, 26.56, 24.77, 24.47, 22.69, 22.60, 21.39, 21.29, 19.73, 19.68, 12.34, 12.25.

**^1^H and ^13^C NMR spectra for d_4_-α-Tocopherol quinone**

^^

^^
