# Supplementary material for: Vitamin E hydroquinone is an endogenous regulator of ferroptosis via redox control of 15-lipoxygenase
Source: PLoS One. 2018 Aug 15;13(8):e0201369. doi: 10.1371/journal.pone.0201369 (PMC6093661; doi:10.1371/journal.pone.0201369)
Supplement: S2 Method — (DOCX) [file pone.0201369.s002.docx]

# **Supporting Information**

## **S2 Method. The synthesis of αTCC – alpha tocopherol carbochroman**

General Chemistry Procedures

The following abbreviations were used in this section:

AcOH – Acetic acid

DCM – Dichloromethane

DME – 1,2-dimethoxyethane

DMF – Dimethyl formamide

DMSO – Dimethyl sulfoxide

LAH – Lithium aluminum hydride

LDA – Lithium diisopropylamide

MeOH – Methanol

MOMCl – Methoxymethyl chloride

MsCl – Methanesulfonyl chloride

MTBE – Methyl *tert*-butyl ether

Pd/C – Palladium on activated carbon

TFA – Trifluoroacetic acid

TFAA - Trifluoroacetic anhydride

THF – Tetrahydrofuran

TLC – Thin layer chromatography

All reagents were obtained from commercial suppliers and used without further purification unless otherwise stated. Compounds **21**-**24** (**Scheme 2**) were analyzed using the following LC/MS methods:

Method A: Merck Millipore Chromolith SpeedROD C_18_ column (50 x 4.6 mm) and a dual gradient run from 5-100% mobile phase B over 12 minutes. Mobile phase A = water (0.1% trifluoroacetic acid (TFA). Mobile phase B = acetonitrile (0.1% TFA).

Method B: Waters Cortecs 2.7µ C_18_ (3.0 mm x 50 mm), Temp: 55 °C; Flow: 1.2 mL/min; Mobile phase: 100% water with 0.1 % TFA then 100% acetonitrile with 0.1% TFA, gradient: 5% to 100% B over 4 min, with stay at 100% B for 0.5 min, equilibration to 5% B over 1.5 min).

**SCHEME 2**

**4-Bromo-2,3,6-trimethylphenol (2)**

To a solution of 2,3,6-trimethylphenol (**1**) (112.1 g, 0.823 mol, 1.0 eq) in DCM (600 mL) was added Br_2_ (42.17 mL, 0.823 mol, 1.0 eq) dropwise at room temperature. After addition, the mixture was stirred at room temperature for 16 h under nitrogen atmosphere. HPLC analysis of the reaction mixture showed full conversion to the desired product. The mixture was quenched with 1 M NaHCO_3_ solution (500 mL). The mixture was extracted with DCM and water. The organic phase was washed with brine, dried over anhydrous Na_2_SO_4_ and concentrated under reduced pressure to afford crude 4-bromo-2,3,6-trimethylphenol (**2**) as an off-white solid that was used without further purification.

**1-Bromo-4-methoxy-2,3,5-trimethylbenzene (3)**

To a solution of 4-bromo-2,3,6-trimethylphenol (**2**) (151.1 g, 0.70 mol, 1.0 eq) and KOH (78.78 g, 1.40 mol, 2.0 eq) in DMSO (700 mL) was added MeI (149.5 g, 1.05 mol, 1.5 eq) dropwise at 0 °C. After addition, the mixture was allowed to warm to room temperature and stirred for 3 h under nitrogen atmosphere. TLC analysis of the reaction mixture showed full conversion to the desired product. Then the mixture was poured into water (1.5 L) and extracted with ethyl acetate (3 x 500 mL). The combined organic phases were washed with brine (2 x 1 L), dried over anhydrous Na_2_SO_4_ and concentrated under reduced pressure to afford crude 1-bromo-4-methoxy-2,3,5-trimethylbenzene (**3**) as a red oil. This oil was used in the next step without further purification.

**4-Methoxy-2,3,5-trimethylbenzaldehyde (4)**

To a solution of 1-bromo-4-methoxy-2,3,5-trimethylbenzene (**3**) (80.1 g, 0.35 mol, 1.0 eq) in anhydrous THF (350 mL) was added iPrMgCl (2 M in THF, 87.4 mL, 0.175 mol, 0.5 eq) dropwise at 0 °C. After being stirred for 15 min, n-BuLi (2.5 M in hexane, 167.8 mL, 0.42 mol, 1.2 eq) was added at 0 °C. After being stirred for 30 min, DMF (33.22 g, 0.45 mol, 1.3 eq) was added at 0 °C. The mixture was allowed to warm to room temperature and stirred for 16 h under a nitrogen atmosphere. TLC analysis of the reaction mixture showed full conversion to the desired product. The mixture was quenched with saturated NH_4_Cl solution (500 mL) and extracted with ethyl acetate (2 x 500 mL). The combined organic phases were washed with brine (3 x 500 mL), dried over anhydrous Na_2_SO_4_ and concentrated under reduced pressure to afford crude 4-methoxy-2,3,5-trimethylbenzaldehyde (**4**) as a red oil. This material was carried forward without further purification.

**4-(4-Methoxy-2,3,5-trimethylphenyl)-3-(methoxycarbonyl)but-3-enoic acid (6)**

To a stirring solution of tBuOK (1 M in tBuOH, 336.6 mL, 0.34 mol, 1.2 eq) was added a mixture of 4-methoxy-2,3,5-trimethylbenzaldehyde (**4**) (50 g, 0.28 mol, 1.0 eq) and dimethyl succinate (**5**) (49.2 g, 0.34 mol, 1.2 eq) in tBuOH (50 mL). After addition, the mixture was stirred at room temperature for 2 h under a nitrogen atmosphere. TLC analysis of the reaction mixture showed full conversion to the desired product. Then the mixture was poured into water (500 mL) and extracted with MTBE (2 x 500 mL). The combined organic phases were acidified with HCl to pH = 2-3 and extracted with ethyl acetate (2 x 500 mL). The combined organic phases were dried over anhydrous Na_2_SO_4_ and concentrated under reduced pressure. The residue was purified by silica gel chromatography to afford 4-(4-methoxy-2,3,5-trimethylphenyl)-3-(methoxycarbonyl)but-3-enoic acid (**6**) (28.1 g, 32%) as a yellow solid. ^1^H NMR (400 MHz, CDCl_3_) δ 7.80 (s, 1H), 6.90 (s, 1H), 3.65 (s, 3H), 3.50 (s, 3H), 3.80 (s, 2H), 2.20 (d, 6H), 2.10 (s, 3H).

**4-Methoxy-3-(4-methoxy-2,3,5-trimethylbenzyl)-4-oxobutanoic acid (7)**

To a solution of 4-(4-methoxy-2,3,5-trimethylphenyl)-3-(methoxycarbonyl)but-3-enoic acid (**6**) (55 g, 0.19 mol, 1.0 eq) in MeOH (600 mL) was added 10% Pd/C (3.5 g, 6% w/w) in a sealed tube under nitrogen atmosphere. The reaction mixture was transferred to a Parr^®^ Shaker. The mixture was then shaken at 70 psi hydrogen pressure for 6 h. HPLC analysis of the reaction mixture showed full conversion to the desired product. The mixture was filtered and concentrated under reduced pressure to afford crude 4-methoxy-3-(4-methoxy-2,3,5-trimethylbenzyl)-4-oxobutanoic acid (**7**) (51 g, 92%) as a yellow solid. This was used without further purification.

**Methyl 6-methoxy-5,7,8-trimethyl-4-oxo-1,2,3,4-tetrahydronaphthalene-2-carboxylate (8)**

To a solution of 4-methoxy-3-(4-methoxy-2,3,5-trimethylbenzyl)-4-oxobutanoic acid (**7**) (31 g, 0.10 mol, 1.0 eq) in TFA (300 mL) was added TFAA (47.8 mL, 0.32 mol, 3.15 eq) dropwise at 0 °C. After addition, the mixture was allowed to warm to room temperature and stirred for 16 h under nitrogen atmosphere. TLC analysis of the reaction mixture showed full conversion to the desired product. Then the mixture was concentrated under reduced pressure. The residue was poured into ice-cold water and basified with saturated NaHCO_3_ solution to pH = 8-9. The mixture was extracted with ethyl acetate (3 x 200 mL). The combined organic phases were washed with brine (2 x 1 L), dried over anhydrous Na_2_SO_4_ and concentrated under reduced pressure. The residue was purified by silica gel chromatography to afford crude methyl 6-methoxy-5,7,8-trimethyl-4-oxo-1,2,3,4-tetrahydronaphthalene-2-carboxylate (**8**) (37 g). The residue was treated with petroleum ether:ethyl acetate =10:1 and stirred for 15 min. The mixture was filtered and the filtered cake was washed with petroleum ether (10 mL) to afford pure methyl 6-methoxy-5,7,8-trimethyl-4-oxo-1,2,3,4-tetrahydronaphthalene-2-carboxylate (8) (26.3 g, 55%) as a red solid. ^1^H NMR (400 MHz, CDCl_3_) δ 3.75 (s, 3H), 3.65 (s, 3H), 3.20 (m, 1H), 3.08 (m, 1H), 2.95 (m, 1H), 2.87 (m, 1H), 2.70 (m, 1H), 2.70 (s, 3H), 2.30 (s, 3H), 2.22 (s, 3H).

**Methyl 6-methoxy-5,7,8-trimethyl-1,2,3,4-tetrahydronaphthalene-2-carboxylate (9)**

To a solution of methyl 6-methoxy-5,7,8-trimethyl-4-oxo-1,2,3,4-tetrahydronaphthalene-2-carboxylate (**8**) (12.5 g, 45.2 mmol, 1.0 eq) in AcOH (100 mL) was added 10% Pd/C (2.5 g, 20% w/w) under nitrogen atmosphere. The mixture was charged with hydrogen 3 times and stirred at 70 °C for 20 h. HPLC analysis of the reaction mixture showed full conversion to the desired product. The mixture was then filtered and concentrated under reduced pressure to afford crude methyl 6-methoxy-5,7,8-trimethyl-1,2,3,4-tetrahydronaphthalene-2-carboxylate (**9**) as a white solid. This was used without further purification.

**Methyl 6-methoxy-2,5,7,8-tetramethyl-1,2,3,4-tetrahydronaphthalene-2-carboxylate (10)**

To a solution of methyl 6-methoxy-5,7,8-trimethyl-1,2,3,4-tetrahydronaphthalene-2-carboxylate (**9**) (13 g, 49.6 mmol, 1.0 eq) in anhydrous THF (130 mL) was added LDA (2 M in THF, 42.2 mL, 84.3 mmol, 1.7 eq) dropwise at -78 °C. After stirring for 1 h, MeI (9.8 g, 69.4 mmol, 1.4 eq) was added. The mixture was stirred at -78 °C for an additional 1 h under nitrogen atmosphere. TLC analysis of the reaction mixture showed full conversion to the desired product. The mixture was quenched with saturated NH_4_Cl solution (500 mL) at 0 °C. The mixture was extracted with DCM (3 x 30 mL). The combined organic phases were washed with brine (2 x 60 mL), dried over anhydrous Na_2_SO_4_ and concentrated under reduced pressure. The residue was purified by silica gel chromatography to afford methyl 6-methoxy-2,5,7,8-tetramethyl-1,2,3,4-tetrahydronaphthalene-2-carboxylate (**10**) (11.3 g, 82%) as a white solid. ^1^H NMR (400 MHz, CDCl_3_) δ 3.72 (s, 3H), 3.65 (s, 3H), 2.95 (dd, 1H), 2.81-2.56 (m, 4H). 2.23 (s, 3H), 2.19 (s, 6H), 1.76 (m, 2H).

**Methyl 6-hydroxy-2,5,7,8-tetramethyl-1,2,3,4-tetrahydronaphthalene-2-carboxylate (11)**

To a solution of methyl 6-methoxy-2,5,7,8-tetramethyl-1,2,3,4-tetrahydronaphthalene-2-carboxylate (**10**) (15.2 g, 55.1 mmol, 1.0 eq) in DCM (100 mL) was added BBr_3_ (2 M in DCM, 137.5 mL, 0.275 mmol, 5.0 eq) at 0 °C. After addition, the mixture was allowed to warm to room temperature and stirred for 2 h under nitrogen atmosphere. TLC analysis of the reaction mixture showed full conversion to the desired product. Then the mixture was quenched carefully with MeOH (150 mL) at 0 °C. The mixture was extracted with DCM and water. The combined organic phases were dried over anhydrous Na_2_SO_4_ and concentrated under reduced pressure to afford methyl 6-hydroxy-2,5,7,8-tetramethyl-1,2,3,4-tetrahydronaphthalene-2-carboxylate (**11**) as a white solid. This material was used without further purification.

**Methyl 6-(methoxymethoxy)-2,5,7,8-tetramethyl-1,2,3,4-tetrahydronaphthalene-2-carboxylate (12)**

To a solution of methyl 6-hydroxy-2,5,7,8-tetramethyl-1,2,3,4-tetrahydronaphthalene-2-carboxylate (**11**) (12.1 g, 46.2 mmol, 1.0 eq) in anhydrous THF (120 mL) was added 60% NaH (5.54 g, 0.139 mol, 3.0 eq) at 0 °C. After being stirred for 0.5 h, MOMCl (7.4 g, 92.3 mmol, 2.0 eq) was added. The mixture was allowed to warm to room temperature and stirred for 1 h under nitrogen atmosphere. TLC analysis of the reaction mixture showed full conversion to the desired product. Then the mixture was quenched with saturated NH_4_Cl solution (500 mL) at 0 °C and extracted with ethyl acetate (3 x 500 mL). The combined organic phases were washed with brine (3 x 500 mL), dried over anhydrous Na_2_SO_4_ and concentrated under reduced pressure to afford methyl 6-(methoxymethoxy)-2,5,7,8-tetramethyl-1,2,3,4-tetrahydronaphthalene-2-carboxylate (**12**) as a brown oil. **12** was used crude in the next step.

**(6-(Methoxymethoxy)-2,5,7,8-tetramethyl-1,2,3,4-tetrahydronaphthalen-2-yl)methanol (13)**

To a mixture of LAH (5.96 g, 0.16 mol, 3.0 eq) in anhydrous THF (60 mL) was added a solution of methyl 6-(methoxymethoxy)-2,5,7,8-tetramethyl-1,2,3,4-tetrahydronaphthalene-2-carboxylate (**12**) (16 g, 52.3 mmol, 1.0 eq) in anhydrous THF (100 mL) at 0 °C. The mixture was stirred at 0 °C for 1 h under nitrogen atmosphere. TLC analysis of the reaction mixture showed full conversion to the desired product. Then the mixture was quenched with water (5 mL) and 15% NaOH (5 mL). The mixture was dried over anhydrous MgSO_4_ and filtered. The filtered cake was washed with THF (50 mL). The filtrate was concentrated under reduced pressure and the residue was purified by silica gel chromatography to afford (6-(methoxymethoxy)-2,5,7,8-tetramethyl-1,2,3,4-tetrahydronaphthalen-2-yl)methanol (**13**) (11.62 g, 58%) as a white solid. ^1^H NMR (400 MHz, CDCl_3_) δ 4.89 (s, 2H), 3.63 (s, 3H), 3.45 (q, *J* = 8.0 Hz, 2H), 2.65-2.61 (m, 2H), 2.50-2.46 (m, 1H), 2.36-2.32 (m, 1H), 2.23-2.12 (m, 11H), 1.67-1.58 (m, 1H), 1.55-1.53 (m, 1H), 0.98 (s, 3H).

**SCHEME 3**

**(6-(Methoxymethoxy)-2,5,7,8-tetramethyl-1,2,3,4-tetrahydronaphthalen-2-yl)methyl methanesulfonate (14)**

(6-(methoxymethoxy)-2,5,7,8-tetramethyl-1,2,3,4-tetrahydronaphthalen-2-yl)methanol (**13**) (9.75 g, 35.0 mmol) was added to a 1000 mL round bottom flask fitted with a stir bar and nitrogen bubbler. DCM (300 mL) was added and the resulting solution was cooled in an ice/water bath. Et_3_N (9.8 mL, 70 mmol) was added followed by dropwise addition of MsCl (4.1 mL, 53 mmol). The clear solution was stirred at ice bath temperature for 30 min. Upon reaction completion, NH_4_Cl (20 wt% in water, 150 mL) was added. The resulting suspension was stirred at room temperature for 10 min. The phases were separated. The organic phase was washed with brine (150 mL) and dried over Na_2_SO_4_. The solvent was removed *in vacuo*. The pale-yellow oil was purified by chromatography on a silica gel column eluted with a 0→40% gradient of acetone/hexane over 8 column volumes. A quantitative yield of the title compound was achieved (12.67 g, 35 mmol) as a cloudy colorless oil. ^1^H NMR (400 MHz, DMSO-*d_6_*) δ 4.82 (s, 2H), 4.00 (d, *J* = 1.9 Hz, 2H), 3.50 (s, 3H), 3.18 (s, 3H), 2.58 (m, 2H), 2.48 (d, *J* = 16.8 Hz, 2H), 2.37 (d, *J* = 16.8 Hz, 2H), 2.14 (s, 3H), 2.09 (s, 3H), 2.05 (s, 3H), 1.60 (m, 2H), 0.96 (s, 3H). LC-MS (APCI + ESI) m/z (ion): 357 (M+H)^+^.

**2-(Bromomethyl)-6-(methoxymethoxy)-2,5,7,8-tetramethyl-1,2,3,4-tetrahydronaphthalene (15)**

(6-(methoxymethoxy)-2,5,7,8-tetramethyl-1,2,3,4-tetrahydronaphthalen-2-yl)methyl methanesulfonate (**14**)( 12.45 g, 34.9 mmol) and tetrabutylammonium bromide (113.27 g, 351 mmol) were weighed into a 1000 mL round bottom flask fitted with a stir bar, reflux condenser and nitrogen bubbler. Diglyme (300 mL) was added and the mixture was stirred at 100 °C for 67 h. All the solids dissolved to give a clear pale-yellow solution at around 80 °C. The solution became a light orange color over the course of the reaction. Upon reaction completion, the hot reaction mixture was poured into a 2 L separatory funnel. Washed reaction flask with 600 mL of water and 600 mL of heptane and added these washes to the separatory funnel. The organic layer was separated and the aqueous layer was back extracted with heptane (2 x 300 mL). The combined organics were then washed with water (300 mL), brine (300 mL) and dried over Na_2_SO_4_. The solvent was removed *in vacuo* to obtain a clear amber liquid. This residue was purified by chromatography on a silica gel column eluted with a 0→100% gradient of DCM/hexane over 15 column volumes. 11.11 g of impure product was obtained as a clear colorless oil. This was purified a second time by chromatography on a silica gel column eluted with isocratic 1:9:90 acetone:DCM:heptane. The title compound was obtained as a colorless oil (10.48 g,30.7 mmol, 88%). ^1^H NMR (400 MHz, C_6_D_6_) δ 4.73 (s, 2H), 3.33 (s, 3H), 3.05 (d, *J* = 10.0 Hz, 1H), 2.96 (d, *J* = 10.0 Hz, 1H), 2.45 – 2.22 (m, 4H), 2.18 (s, 3H), 2.10 (s, 3H), 1.90 (s, 3H), 1.52 (dt, *J* = 13.4, 6.8 Hz, 1H), 1.33 (dt, *J* = 13.2, 6.7 Hz, 1H), 0.89 (s, 3H). LC-MS (APCI + ESI) m/z (ion): 340 (M+H)^+^, 342 (M+H)^+^

**((6-(Methoxymethoxy)-2,5,7,8-tetramethyl-1,2,3,4-tetrahydronaphthalen-2-yl)methyl)magnesium bromide (16)**

To ensure anhydrous reaction conditions, all glassware was dried in an oven at 120 °C overnight. All syringes, needles, septa, and filters were dried under vacuum over CaSO_4_ for a minimum of 24 hours. To a 250-ml three-necked round bottom flask fitted with a Teflon-coated stir bar, stopper, septum, and reflux condenser connected to an argon inlet was added freshly cut potassium (2.60 g, 66.5 mmol), anhydrous magnesium chloride (3.52 g, 37.0 mmol), anhydrous potassium iodide (5.00 g, 30.1 mmol) and THF (50 mL). The mixture was stirred vigorously and heated to reflux for 2 hours.

A solution of 2-(bromomethyl)-6-(methoxymethoxy)-2,8-dimethyl-1,2,3,4-tetrahydronaphthalene (**15**) (6.30 g, 18.5 mmol) dissolved in 25 mL of anhydrous THF was added to the refluxing magnesium suspension dropwise over 30 min using a syringe pump. Stirring was continued at reflux for 60 min after the addition was complete. The reaction flask was then transferred to a sonic bath and sonicated at room temperature for 10 min. The resulting suspension was filtered through a 0.2 µM PTFE filter into a clean, oven dried 250 mL round bottom flask under argon. The residual solids were washed with 2 x 10 mL of anhydrous THF, filtered and combined. 110 mL of a clear amber solution was obtained. The solution was titrated at 0.12 M using the method of Krasovskiy and used without purification. (Krasovskiy, A. and Knochel, P. Convenient Titration Method for Organometallic Zinc, Magnesium, and Lanthanide Reagents. *SYNTHESIS* **38**, 890–891 (2006)).

**6-(Methoxymethoxy)-2,5,7,8-tetramethyl-2-(4,8,12-trimethyltridecyl)-1,2,3,4-tetrahydronaphthalene (18)**

Copper(II) chloride (34.5 mg, 0.256 mmol) was weighed into an oven-dried 500 mL round bottom flask fitted with a stir bar and septum. To this was added a 15 wt% solution of 1,3-butadiene in hexane(2.7 mL, 5.2 mmol). Anhydrous THF (50 mL) was added and the suspension was sonicated for 5 min to dissolve the CuCl_2_ and obtain a clear yellow solution. This was cooled in an ice/water bath. To the cooled solution was added 1-bromo-3,7,11-trimethyl-dodecane (**17**) (1.42 g, 5.0 mmol). After stirring for 15 min ((6-(methoxymethoxy)-2,5,7,8-tetramethyl-1,2,3,4-tetrahydronaphthalen-2-yl)methyl)magnesium bromide (**16**) (45 mL, 5.4 mmol, 0.12 M solution in THF) was added dropwise over 15 min using a syringe pump. The reaction mixture turned red then slightly cloudy medium brown, then clear light brown. The ice bath was removed and the reaction mixture was stirred at room temperature overnight. At this time, the reaction was quenched with NH_4_Cl (100 mL of 20 wt% in water). The organic phase was removed *in vacuo*. The remaining aqueous mixture was extracted with 200 mL of heptane and washed with 100 mL of 20 wt% NH_4_Cl in water, 100 mL of water, and 100 mL of brine. The organic solvent was dried over Na_2_SO_4_ and concentrated to dryness. The resulting pale-yellow oil was purified by chromatography on a silica gel column eluted with a 0→100% gradient of DCM/hexane over 12 column volumes. The title compound was obtained as a colorless oil (1.19 g, 2.52 mmol, 50%). ^1^H NMR (400 MHz, C_6_D_6_) δ 4.79 (d, *J* = 0.8 Hz, 2H), 3.38 (s, 3H), 2.55 (m, 2H), 2.47 (d, *J* = 16.4 Hz, 1H), 2.38 (d, *J* = 16.4 Hz, 1H), 2.26 (s, 3H), 2.23 (s, 3H), 2.05 (s, 3H), 1.58 – 1.23 (m, 18H), 1.22 – 1.14 (m, 5H), 1.01 – 0.86 (m, 15H). LC-MS (APCI + ESI) m/z (ion): 473 (M+H)^+^

**1,3,4,6-Tetramethyl-6-(4,8,12-trimethyltridecyl)-5,6,7,8-tetrahydronaphthalen-2-ol (19)**

6-(methoxymethoxy)-2,5,7,8-tetramethyl-2-(4,8,12-trimethyltridecyl)-1,2,3,4-tetrahydronaphthalene (**18**)(1.03 g, 2.18 mmol) was added to a 100 mL round bottom flask fitted with a stir bar and loose cap. To this was added DME (20 mL) and MeOH (20 mL) containing 0.90 mL (11 mmol) of 12 M hydrochloric acid. The reaction mixture was stirred at 50 °C for 60 min and then quenched with 20 mL of 20 wt% NH_4_Cl in water. The organic solvent was removed *in vacuo*. The aqueous solution was extracted with 40 mL of heptane. The organic phase was separated and washed with 2 x 20 mL of water and 20 mL of brine. This was dried over Na_2_SO_4_ and concentrated to dryness to obtain a yellow oil. The residue was purified by chromatography on a silica gel column eluted with a 0→75% gradient of DCM/hexane over 9 column volumes. The title compound was obtained as a colorless oil (616 mg, 1.44 mmol, 66%). ^1^H NMR (400 MHz, C_6_D_6_) δ 4.04 (s, 1H), 2.56 (t, *J* = 5.8 Hz, 2H), 2.46 (d, *J* = 16.2 Hz, 1H), 2.37 (d, *J* = 16.1 Hz, 1H), 2.05 (d, *J* = 2.4 Hz, 6H), 1.96 (s, 3H), 1.58 – 1.23 (m, 18H), 1.22 – 1.13 (m, 5H), 1.04 – 0.86 (m, 15H). LC-MS (APCI + ESI) m/z (ion): 429 (M+H)^+^
