# Supplementary material for: Vitamin E hydroquinone is an endogenous regulator of ferroptosis via redox control of 15-lipoxygenase
Source: PLoS One. 2018 Aug 15;13(8):e0201369. doi: 10.1371/journal.pone.0201369 (PMC6093661; doi:10.1371/journal.pone.0201369)
Supplement: S3 Method — (DOCX) [file pone.0201369.s003.docx]

# **Supporting Information**

## **S3 Method. Cellular quantification of alpha-tocopherol carbochroman (αTCC)**

Due to poor ionization of **αTCC**, the succinate capping method was used for quantification of the succinate ester derivative formed by cell lysis with 100 mg/mL succinic anhydride in 95:5 (acetonitrile:triethylamine).

Q7 cells were cultured in standard culture medium for 18 hours prior to compound addition. After the specified treatment time, the culture medium was removed and adherent cells were washed with PBS. The wash was removed and adherent cells were harvested by adding 1 mL of 100 mg/mL succinic anhydride in 95:5 (acetonitrile:triethylamine) followed by immediate cell scrapping. After cell scrapping, samples were transferred to a 1.5 mL Eppendorf tube, vortexed for 15 seconds, and kept on wet ice until all samples were harvested. Samples were vortexed again for 15 seconds and centrifuged in a Thermo Sorvall legend XFR centrifuge at 12,000 rpm, 4 °C, for 3 minutes.

Following centrifugation, samples were protein-precipitated by addition of 3 volumes of acetonitrile. Following precipitation, samples were vortexed for 30 seconds and centrifuged in a Thermo Sorvall legend XFR centrifuge at 4000 rpm, 4 °C, for 10 minutes. Subsequently, 1 volume of supernatant was transferred to a 384-well injection plate and diluted with 1 volume of mobile phase A and pipette mixed. Internal standard was added to the 384-well plate and pipette mixed. The injection plate was vortexed and centrifuged for a final time at 4000 rpm, 4 °C, for 10 minutes and subsequently placed in a chilled Shimadzu SIL-30AC autosampler at 4 °C for LCMS/MS analysis.

Samples were analyzed using a developed method on a Shimadzu 30AD using micro-flow with a Shimadzu SIL-30AC autosampler, and coupled to an AB Sciex 6500+ QTRAP mass spectrometer. The MS/MS instrument was operated in positive ESI mode. Electrospray conditions for the µLC-ESI-MS/MS method were optimized and were as follows: Ion spray voltage was set to 5500V, temperature of 250 °C, curtain gas of 20, CAD gas of 10, and ion source gas 1 and 2 of 15 and 60 psi respectively. Analyzer parameters were optimized for each compound using a combination of manual tuning and compound optimization. **αTCC** was measured as **αTCC**-succinate. The MRM transition used to measure αTCC succinate was 529.334→76.900.

A Phenomenex Kinetex C_18_ (2.1 x 50 mm, 1.3 µm) column using a reverse phase gradient mobile phase method was used for chromatographic separation. The composition of mobile phase A was 0.1% formic acid in water. The composition of mobile phase B was 3:1 (0.1% formic acid in (acetonitrile: isopropyl alcohol)). Mobile Phase B was ramped from 30% to 98% over 7 minutes. The total run time was 8 minutes. Data were analyzed using Sciex Analyst Chromatography software, version 1.6.2 and Sciex MultiQuant software. The standard curve equation (y=mx +b) is generated from the calibration standards with weighted regression.
