# Supplementary material for: Vitamin E hydroquinone is an endogenous regulator of ferroptosis via redox control of 15-lipoxygenase
Source: PLoS One. 2018 Aug 15;13(8):e0201369. doi: 10.1371/journal.pone.0201369 (PMC6093661; doi:10.1371/journal.pone.0201369)
Supplement: S1 Fig — (DOCX) [file pone.0201369.s004.docx]

# **Supporting Information**

## **S1 Fig. EPR spectrum of the radical formed upon oxidation of αTCC**

⍺TCC^●^ spectrum recorded with optimized EPR settings reveals that the unpaired electron is delocalized over an aromatic ring with multiple ^1^H hyperfine splittings observed, indicating that the radical generated is analogous to the ⍺T^●^ (ref: Matsuo M, Matsumoto S. Electron Spin Resonance Spectra of the Chromanoxyl Radicals Derived from Tocopherols (Vitamin E) and Their Related Compounds. Lipids 1983;18: 81-86. doi:10.1007/BF02534695).
