# Supplementary material for: Vitamin E hydroquinone is an endogenous regulator of ferroptosis via redox control of 15-lipoxygenase
Source: PLoS One. 2018 Aug 15;13(8):e0201369. doi: 10.1371/journal.pone.0201369 (PMC6093661; doi:10.1371/journal.pone.0201369)
Supplement: S1 Table — (DOCX) [file pone.0201369.s005.docx]

# **Supporting Information**

## **S1 Table. The metabolites measured in culture medium during ferroptosis by LC-MS/MS.**

The 19 Arachidonic acid metabolites measured in culture medium during ferroptosis by LC-MS/MS:

| 5-HETE |
| --- |
| 5-oxoETE |
| 5-HpETE |
| 12-HETE |
| 12-oxoETE |
| 12-HpETE |
| 15-HETE |
| 15-oxoETE |
| 15-HpETE |
| 8-HETE |
| 9-HETE |
| 11-HETE |
| Arachidonic Acid |
| Thromboxane B2 |
| 12-HHTrE |
| 6-keto-PGF1a |
| PGF2a |
| PGE2 |
| PGD2 |
